# Effectiveness of interventions to improve drinking water, sanitation, and handwashing with soap on risk of diarrhoeal disease in children in low-income and middle-income settings: a systematic review and meta-analysis

**DOI:** 10.1016/S0140-6736(22)00937-0

**Published:** 2022-07-02

**Authors:** Jennyfer Wolf, Sydney Hubbard, Michael Brauer, Argaw Ambelu, Benjamin F Arnold, Robert Bain, Valerie Bauza, Joe Brown, Bethany A Caruso, Thomas Clasen, John M Colford, Matthew C Freeman, Bruce Gordon, Richard B Johnston, Andrew Mertens, Annette Prüss-Ustün, Ian Ross, Jeffrey Stanaway, Jeff T Zhao, Oliver Cumming, Sophie Boisson

**Affiliations:** a Department of Environmental, Climate Change and Health, WHO, Geneva, Switzerland; b Gangarosa Department of Environmental Health, Rollins School of Public Health, Emory University, Atlanta, GA, USA; c The Hubert Department of Global Health, Rollins School of Public Health, Emory University, Atlanta, GA, USA; d Institute for Health Metrics and Evaluation, University of Washington, Seattle, WA, USA; e School of Population and Public Health, University of British Columbia, Vancouver, BC, Canada; f Department of Disease Control, Faculty of Infectious Tropical Disease, London School of Hygiene & Tropical Medicine, London, UK; g Department of Environmental Health Sciences and Technology, Faculty of Public Health, Jimma University, Jimma, Ethiopia; h Division of Epidemiology and Biostatistics, School of Public Health, University of California, Berkeley, CA, USA; i UNICEF Middle East and North Africa, Amman, Jordan; j Department of Environmental Sciences and Engineering, Gillings School of Global Public Health, University of North Carolina at Chapel Hill, NC, USA

## Abstract

**Background:**

Estimates of the effectiveness of water, sanitation, and hygiene (WASH) interventions that provide high levels of service on childhood diarrhoea are scarce. We aimed to provide up-to-date estimates on the burden of disease attributable to WASH and on the effects of different types of WASH interventions on childhood diarrhoea in low-income and middle-income countries (LMICs).

**Methods:**

In this systematic review and meta-analysis, we updated previous reviews following their search strategy by searching MEDLINE, Embase, Scopus, Cochrane Library, and BIOSIS Citation Index for studies of basic WASH interventions and of WASH interventions providing a high level of service, published between Jan 1, 2016, and May 25, 2021. We included randomised and non-randomised controlled trials conducted at household or community level that matched exposure categories of the so-called service ladder approach of the Sustainable Development Goal (SDG) for WASH. Two reviewers independently extracted study-level data and assessed risk of bias using a modified Newcastle-Ottawa Scale and certainty of evidence using a modified Grading of Recommendations, Assessment, Development, and Evaluation approach. We analysed extracted relative risks (RRs) and 95% CIs using random-effects meta-analyses and meta-regression models. This study is registered with PROSPERO, CRD42016043164.

**Findings:**

21 290 records were identified from the search, of which 124 studies were included, providing 83 water (62 616 children), 20 sanitation (40 799 children), and 41 hygiene (98 416 children) comparisons. Compared with untreated water from an unimproved source, risk of diarrhoea was reduced by up to 50% with water treated at point of use (POU): filtration (n=23 studies; RR 0·50 [95% CI 0·41–0·60]), solar treatment (n=13; 0·63 [0·50–0·80]), and chlorination (n=25; 0·66 [0·56–0·77]). Compared with an unimproved source, provision of an improved drinking water supply on premises with higher water quality reduced diarrhoea risk by 52% (n=2; 0·48 [0·26–0·87]). Overall, sanitation interventions reduced diarrhoea risk by 24% (0·76 [0·61–0·94]). Compared with unimproved sanitation, providing sewer connection reduced diarrhoea risk by 47% (n=5; 0·53 [0·30–0·93]). Promotion of handwashing with soap reduced diarrhoea risk by 30% (0·70 [0·64–0·76]).

**Interpretation:**

WASH interventions reduced risk of diarrhoea in children in LMICs. Interventions supplying either water filtered at POU, higher water quality from an improved source on premises, or basic sanitation services with sewer connection were associated with increased reductions. Our results support higher service levels called for under SDG 6. Notably, no studies evaluated interventions that delivered access to safely managed WASH services, the level of service to which universal coverage by 2030 is committed under the SDG.

**Funding:**

WHO, Foreign, Commonwealth & Development Office, and National Institute of Environmental Health Sciences.

## Introduction

Safe and reliable drinking water, sanitation that separates excreta from human contact, and handwashing with soap at key times, such as after potential faecal contact and before eating or preparing food, have positive effects on health and social and economic wellbeing.^1^, ^2^, ^3^, ^4^, ^5^ However, quantification of the global burden of disease related to improvements to water, sanitation, and hygiene (WASH) is limited by the challenge of securing reliable estimates of risk from diverse settings, conditions, and populations.^3^, ^6^ The UN's Sustainable Development Goals (SDGs) include dedicated WASH targets (SDGs 6.1 and 6.2), which explicitly emphasise equity, address hygiene, and call for higher levels of drinking water and sanitation services for all,^7^ and are linked to all other SDGs.^8^

**Research in context**

**Evidence before this study**

Previous systematic reviews have consistently found that interventions improving drinking water, sanitation, and hygiene (WASH) reduce incidence of childhood diarrhoea. However, these analyses have often not considered intervention types extending beyond basic WASH services, such as drinking water delivered on premises, of high quality, or that is continuously available, or sanitation that safely disposes excreta or removes and safely treats it offsite. We searched Ovid MEDLINE, Embase, Scopus, Cochrane Library, and BIOSIS Citation Index for studies on WASH published between Jan 1, 1970, and May 25, 2021, that matched exposure scenarios informed by the service ladder approach of the Sustainable Development Goal (SDG) targets for WASH. This systematic review contains evidence from 124 WASH interventions.

**Added value of this study**

This systematic review and meta-analysis examines the effect of different types of WASH intervention, matching the transitions envisaged under SDG 6. The study provides updated exposure–response relationships between higher levels of WASH services, such as safely managed drinking water, sewered sanitation, and handwashing with soap, and occurrence of diarrhoea. These new estimates for the effect of different WASH services on diarrhoeal disease provide new information to support policy and investment decisions. They are also essential for up-to-date assessments of the burden of diarrhoeal disease attributable to WASH. Pooled effect sizes for higher level WASH services are important because they allow for more ambitious minimum risk exposure levels, which have a direct influence on the size, accuracy, and relevance of the estimated disease burden attributable to unsafe WASH.

**Implications of all the available evidence**

The effects of WASH interventions on childhood diarrhoea are determined by the level of service achieved. Although a substantial body of evidence is available from interventions of basic WASH services (eg, simple improved drinking water or sanitation, or point-of-use drinking water treatment), evidence from interventions providing higher levels of WASH services remains scarce. Our analysis suggests that WASH services extending beyond basic WASH services are needed to achieve increased benefits on health. Notably, no intervention was identified that delivered access to safely managed WASH services, which are the metrics used to monitor the SDG targets of safe drinking water and adequate sanitation for all by 2030.

According to the WHO–UNICEF Joint Monitoring Programme for Water Supply, Sanitation and Hygiene, in 2020, 2·0 billion people did not have access to safely managed drinking water services, 3·6 billion did not have access to safely managed sanitation services, and 2·3 billion did not have access to handwashing facilities with soap and water at home.^9^ Unsafe WASH is estimated to cause more than 1 million deaths from infectious diseases every year,^6^, ^10^ with a disproportionate burden on children younger than 5 years. These estimates are not disaggregated by sex, despite calls and guidelines to do so in public health^11^, ^12^ and in the WASH sector.^13^

Previous systematic reviews on WASH interventions and diarrhoea provided effect estimates for basic sanitation interventions^3^ or point-of-use (POU) water treatment.^1^ A systematic review focusing on the effects of sewerage on incidence of diarrhoea included intervention and non-intervention studies, and searched the literature from Jan 1, 1966, to Feb 28, 2010.^14^ A systematic review on different types of WASH interventions searched the literature up to early 2016 and found only one study reporting provision of higher-quality piped water and two studies of continuous water supply.^5^

In 2021, a *Lancet* Commission on WASH and health was published.^15^ For the work of the Commission, updated estimates of disease burden attributable to WASH are needed, which require up-to-date estimates for the effect of different WASH exposures on risk of diarrhoea. This process is being done in collaboration between several institutions, with the aim of achieving greater alignment between previously divergent burden of disease estimates.^6^, ^10^ The findings of many interventions providing different levels of WASH services have been published since the last systematic review of WASH interventions.^5^ These results include those from three large, high-quality WASH trials, which led to discussion and questions about the health effects of basic WASH interventions (eg, provision of improved latrines or POU treatment of drinking water).^16^

Frequently updated exposure–response relationships from systematic reviews are also needed for annual reporting of progress towards SDG 3 to ensure health and wellbeing for all, and specifically for target 3.9.2 on WASH-related mortality.^17^ We aimed to provide up-to-date estimates for the effect of both basic WASH interventions and WASH interventions that deliver higher levels of service, such as safely managed drinking water and sanitation, on risk of childhood diarrhoea in low-income and middle-income countries (LMICs).

## Methods

### Search strategy and selection criteria

This systematic review and meta-analysis was conducted across multiple institutions, including WHO and the Institute for Health Metrics and Evaluation. On Sept 25, 2020, and again on June 23, 2021, we searched Ovid MEDLINE, Embase, Scopus, Cochrane Library, and BIOSIS Citation Index (Web of Knowledge) using both key words and Medical Subject Headings terms. The search strategy and a full list of search terms are provided in appendix 1 (p 1). This search included literature published between Jan 1, 2016, and May 25, 2021. Including the results from two previous systematic reviews, this systematic review covered studies published from Jan 1, 1970.^5^, ^18^ Studies were included if they were published in a peer-reviewed scientific journal in English or French, or had been assessed according to transparent criteria for methodological quality in a previously conducted systematic review.

**Figure 1:**
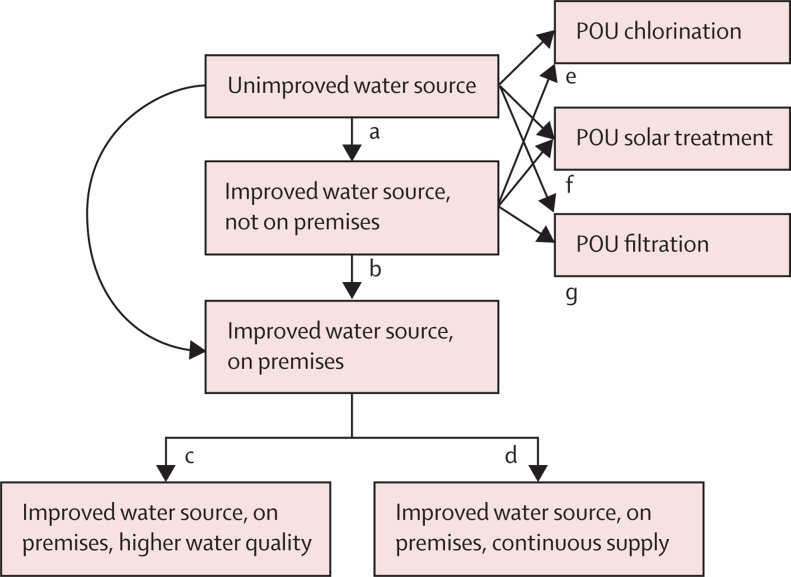
Exposure scenario for drinking water services POU=point-of-use.

**Figure 2:**
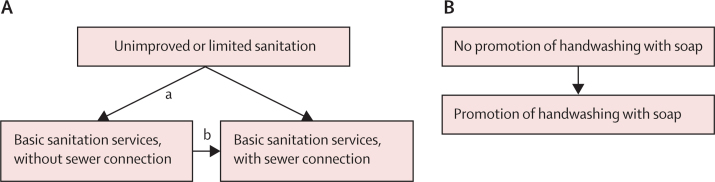
Exposure scenario for sanitation (A) and hygiene (B) services

Eligible study designs were randomised studies, including individual and cluster-randomised controlled trials, and non-randomised and quasi-randomised studies, including those with cohort, case-control, cross-sectional, controlled before-and-after, and interrupted time-series designs. Only studies of interventions were eligible for this systematic review.^19^ Eligible interventions were those improving household or community water supply, sanitation, and hygiene services, and promotion or provision of POU water treatment or promotion of handwashing with soap alone or in combination with broader hygiene promotion that matched our exposure scenarios (Figure 1, Figure 2). To meet the eligibility criteria, interventions needed to be compared against a control group that either did not receive the intervention or received a different intervention type or placebo.

We included single and combined water and sanitation interventions that reported relative risk (RR) estimates and confidence intervals, or the relevant data to calculate them. Interventions including both a drinking water and sanitation component matching our exposure scenarios (Figure 1, Figure 2) were included in the water and sanitation analyses. Given that handwashing interventions are often added as an additional component to water and sanitation interventions, studies included in the hygiene analysis needed to report effect estimates separately for the handwashing intervention (ie, a separate handwashing group) or have the handwashing intervention as the main intervention component. We only included handwashing interventions that promoted handwashing or improved access to handwashing facilities and materials; and we excluded interventions exclusively concerning hand sanitisers (eg, alcohol-based hand gels). Interventions promoting handwashing with soap that also involved messages on drinking water and sanitation behaviour or access were included in the hygiene analysis, but not in the water or sanitation analyses.^20^, ^21^, ^22^ For water and sanitation interventions, we restricted study location to households in LMICs^23^ and in low-income settings in high-income countries.^24^ Handwashing interventions performed in day-care centres or day-care homes for children, and primary schools from high-income countries were included for consistency with other reviews^2^ and because we assumed that these settings represented an increased potential for transmission of faecal pathogens. We excluded studies of WASH interventions in which the study population was considered to be non-representative of the general population with regard to the exposure–outcome relationship of interest (eg, interventions targeting HIV-positive communities, interventions in refugee camps, or hospital patients).

We used Covidence software for systematic reviews (Veritas Health Innovation, Melbourne, VIC, Australia) for data de-duplication throughout full-text review. Two reviewers (JW and SH) screened study titles, abstracts, and full texts. These two reviewers were not involved in any of the included studies as a result of this new search. Any conflicts between the reviewers were resolved through consensus with a third reviewer (SB). The same two reviewers independently extracted data and assessed risk of bias. Any conflicts between reviewers over data extraction and bias assessment were resolved by discussion.

### Data analysis

We extracted summary-level effect estimates on occurrence of diarrhoeal disease, baseline data on study setting, time from intervention implementation to health effect assessment in months, reference WASH service levels, types and number of study population, and type of intervention. We preferably extracted data disaggregated by sex on morbidity from diarrhoea in children younger than 5 years; however, if these data were not available, we included non-disaggregated estimates for all ages (including adults) or older children (aged ≥5 years). Data were extracted using a structured format, which had also been used in the two previous systematic reviews.^5^, ^18^ Study authors were contacted when necessary data were not provided in the reports. We used Covidence for de-duplication of data. If the results of a study were published in both a report and a peer-reviewed journal article, data from the peer-reviewed journal article were extracted in priority. If required data were reported in the report only, these data would be extracted instead.

We extracted adjusted RRs from intention-to-treat analysis in the following order of preference: longitudinal prevalence ratio,^25^ prevalence ratio or risk ratio, (incidence) rate ratio, and odds ratio. When necessary, we calculated RRs and 95% CI from available data. Standard errors of the log RRs were calculated with standard formulae.^26^ Odds ratios were converted to RRs when the risk of the control group was reported.^27^ Risk ratios, prevalence ratios, and rate ratios were combined without conversion. In the case of multiple comparisons within a single study, we included effect estimates from independent subgroups compared with those from separate control groups, but derived a single pairwise comparison for studies without separate control groups. Further detail on multiple comparisons within a single study is provided in appendix 2 (p 3).

Risk of bias in individual studies was assessed by use of a modified Newcastle-Ottawa Scale,^28^ considering seven areas of bias: selection bias, response bias, follow-up bias, misclassification bias, assessment of bias in outcome, measurement of bias in outcome, and bias in analysis. Each study was assigned a score of up to nine, with a higher score indicating a smaller risk of bias (appendix 1 pp 2–4). We assessed the body of evidence by intervention type using a modified Grading of Recommendations, Assessment, Development, and Evaluation (GRADE) approach.^29^ We then used the five GRADE criteria to potentially downgrade the initial rating: risk of bias in individual studies, inconsistency (*I*^2^ >90%), indirectness, imprecision, and publication bias (appendix 2 pp 22–23).

The WASH exposure scenarios (Figure 1, Figure 2) followed definitions and exposure levels of the Joint Monitoring Programme for Water Supply, Sanitation and Hygiene service ladders (appendix 2 p 21) used for SDG monitoring,^9^ and were adapted on the basis of available evidence from intervention studies.

Random-effects meta-analyses were conducted separately for water, sanitation, and handwashing interventions. A random-effects meta-regression model was used to examine transitions according to WASH exposure scenarios, to take account of comparison WASH service levels, and to assess further covariates.

The transitions a to g in figure 1 and a to b in figure 2 present basic parameters, each represented by a covariate in the meta-regression model. All other transitions were coded as combinations of these parameters; for example, the transition from unimproved to improved, on premises, and higher water quality (a + b + c; figure 1) or the transition from unimproved or limited sanitation to basic sanitation services with sewer connection (a + b; figure 2)*.* The model allowed for the indirect estimation of transitions that have not been directly observed, following the ideas of a network meta-analysis.^30^

In addition to these basic parameters, an additional covariate was included in the meta-regression models for water and sanitation to indicate whether or not the intervention was a combined WASH intervention (ie, combined with hygiene or sanitation in water studies or hygiene and water in sanitation studies). We assessed further covariates in separate meta-regression models (appendix 2 pp 13–14).

In the sensitivity analyses, we excluded studies reporting effect estimates for individuals of all ages or children older than 5 years, included results from analyses of survey data with particular matching techniques for comparability with the two previous reviews (appendix 1 p 9),^5^, ^18^ excluded all studies published before 2010, and removed the two sanitation studies that did not account for clustering with the largest effect on diarrhoea (appendix 2 p 14).^31^, ^32^

All statistical analyses were done with Stata (version 14.2). A comparison of methods between the current and previous systematic reviews, including a discussion on adjustment for bias due to non-masking, is provided in appendix 2 (pp 2–3). This systematic review and meta-analysis followed Preferred Reporting Items for Systematic reviews and Meta-Analyses guidelines.^33^ This study is registered with PROSPERO, CRD42016043164.

### Role of the funding source

The funders of the study had no role in study design, data collection, data analysis, data interpretation, or writing of the report.

## Results

**Figure 3:**
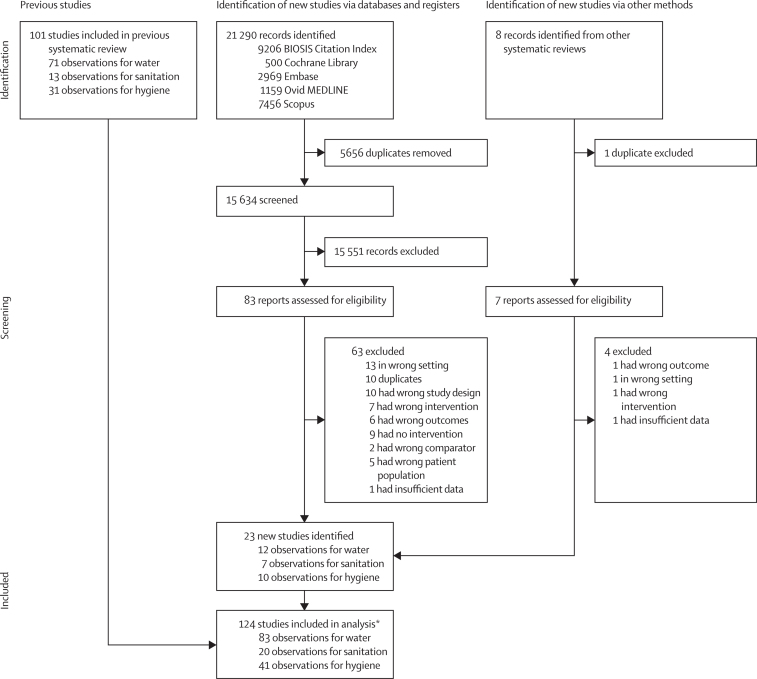
Study selection *Including 78 separate studies for water comparisons, 19 separate studies for sanitation comparisons, and 41 separate studies for hygiene comparisons, as well as 12 studies that provided data on more than one analysis, two of which provided data on all three analyses.

21 290 records were identified from the search, of which 15 634 were screened and 83 assessed for eligibility (figure 3). 23 new studies were added to the previous meta-analyses, including 12 new comparisons for the water analysis, seven for the sanitation analysis, and ten for the hygiene analysis. In total, 124 studies were included in this systematic review, with 83 drinking water comparisons (78 separate studies), 20 sanitation comparisons (19 separate studies), and 41 hygiene comparisons (41 separate studies). These included 12 studies that provided data on more than one analyses, two of which provided data on all three analyses (figure 3). Appendix 1 lists the 23 studies that were added from this review (p 10) and the 101 studies that were added from previous reviews (p 11). The total study population included 62 616 children in the drinking water analysis, 40 799 children in the sanitation analysis, and 98 416 children in the hygiene analysis. Details of individual studies, including effect sizes, randomisation status, setting, and intervention compliance, as well as a list of excluded interventions after full-text review, are provided in appendix 1 (pp 5–8). The pooled estimates from this meta-analysis are compared with those from other reviews in appendix 2 (pp 24–27).

The random-effects meta-analysis of all 83 drinking water comparisons resulted in a pooled RR of 0·68 (95% CI 0·63–0·74) with an *I*^2^ of 92%, indicating considerable heterogeneity of effect estimates between studies. The random-effects meta-regression model of drinking water interventions according to figure 1 indicated that provision of improved drinking water on premises with higher water quality or POU filtration was associated with a larger reduction in risk of diarrhoea than were other intervention types. Compared with untreated water from an unimproved source, diarrhoea risk was reduced by 52% (n=2; 0·48 [0·26–0·87]) with provision of an improved drinking water supply on premises with higher water quality and by up to 50% with water treated at POU: filtration (n=23 studies; RR 0·50 [95% CI 0·41–0·60]), solar treatment (n=13; 0·63 [0·50–0·80]), and chlorination (n=25; 0·66 [0·56–0·77]; table 1). The meta-regression model explained 11% of the between-study variance of drinking water studies. Further examined covariates, including improved sanitation (p=0·35) and rural setting (p=0·37), were not associated with risk of diarrhoeal disease; appendix 2 p 13).

**Table 1:** Results of the meta-regression model for water supply interventions

	**Improved source, not on premises**	**Improved source, on premises**	Improved source, on premises, higher water quality*	**Improved source, on premises, continuous supply**†	**POU chlorination**	**POU solar treatment**	**POU filtration**
**Unimproved**							
RR (95% CI)	0·81 (0·70–0·94)	0·79 (0·60–1·03)	0·48 (0·26–0·87)	0·73 (0·37–1·44)	0·66 (0·56–0·77)	0·63 (0·50–0·80)	0·50 (0·41–0·60)
p value	0·0060	0·076	0·017	0·36	<0·0001	0·0002	<0·0001
**Improved, not on premises**							
RR (95% CI)	..	0·97 (0·75– 1·25)	0·59 (0·32– 1·07)	0·90 (0·46–1·77)	0·81 (0·68–0.95)	0·78 (0·63– 0·96)	0·61 (0·49– 0·75)
p value	..	0·79	0·081	0·75	0·012	0·023	<0·0001
**Improved, on premises**							
RR (95% CI)	..	..	0·61 (0·35– 1·05)	0·93 (0·50– 1·74)	..	..	..
p value	..	..	0·072	0·82	..	..	..

Results are adjusted for combined intervention (RR 0·89 [95% CI 0·74–1·08]). POU=point-of-use. RR=relative risk.

* Based on two studies.^24^, ^34^

† Based on one study.^35^

POU interventions were graded as moderate certainty evidence and all other water interventions as very low certainty evidence (appendix 2 pp 22–24). Only four studies of drinking water (three POU filter studies^36^, ^37^, ^38^ and one POU chlorine study^39^) reported results by sex; thus, our estimates are not disaggregated by sex (appendix 1 pp 5–7). A qualitative description of studies that reported results by sex is provided in appendix 2 (p 16).

Overall, the random-effects meta-analysis of all 20 sanitation comparisons showed a reduction in risk of diarrhoea of 24% (RR 0·76 [95% CI 0·61–0·94]), with an *I*^2^ of 96% (appendix 2 p 10). Results of the random-effects meta-regression model according to figure 2 indicated that provision of basic sanitation services with sewer connection was associated with a 47% reduction (0·53 [0·30–0·93]) in risk of diarrhoea, when tested against unimproved or limited sanitation. Basic sanitation without sewer connection was associated with a 21% reduction (0·79 [0·61–1·03]) in risk of diarrhoea when tested against unimproved or limited sanitation (table 2). The meta-regression model explained 17% of the between-study variance of sanitation studies. Sanitation interventions with a longer follow-up time (≥24 months) were associated with smaller reductions in diarrhoea (p=0·005). There was some evidence that reductions in diarrhoea through sanitation interventions were higher when drinking water was provided from an unimproved source (p=0·10) and the study was non-randomised (p=0·06). No effect was found for other covariates (p>0·10; appendix 2 pp 13–14). Results from the subgroup meta-analysis of sanitation intervention types are provided in appendix 2 (p 10).

**Table 2:** Results of the meta-regression model for sanitation interventions

	**Basic sanitation services, without sewer connection (n=15)**	**Basic sanitation services, with sewer connection (n=5)**
**Unimproved or limited sanitation**		
RR (95% CI)	0·79 (0·61–1·03)	0·53 (0·30–0·93)
p value	0·077	0·030
**Basic sanitation services, without sewer connection**		
RR (95% CI)	..	0·66 (0·41–1·07)
p value	..	0·089

Results are adjusted for combined intervention (RR 0·95 [95% CI 0·65–1·40]). RR=relative risk.

Basic sanitation services without sewer connection were graded as moderate certainty evidence and basic sanitation services with sewer connection were graded as very low certainty evidence mainly due to the non-randomised study designs and high risk of bias in services with sewer connection (appendix 2 pp 22–24). No sanitation studies reported results by sex.

**Figure 4:**
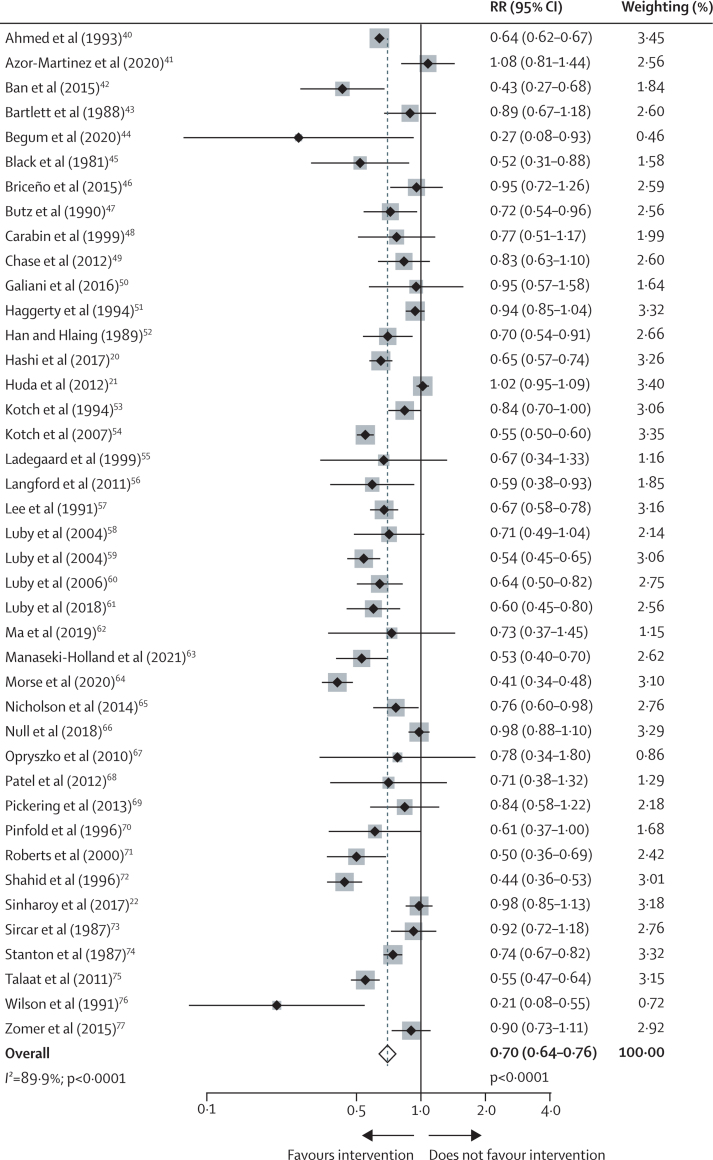
Effects of hygiene interventions on risk of diarrhoea Weights are from the random-effects model. RR=relative risk.

The random-effects meta-analysis of all 41 hygiene comparisons showed a reduction in risk of diarrhoea of 30% (RR 0·70 [95% CI 0·64–0·76]), with an *I*^2^ of 90% (figure 4). Handwashing interventions were graded as moderate certainty evidence (appendix 2 pp 22–24). None of the covariates were associated with risk of diarrhoeal disease in the meta-regression analysis of handwashing interventions (appendix 2 p 14). A forest plot of the subgroup meta-analysis of low-income and middle-income settings versus high-income settings is shown in appendix 2 (p 12). Only one handwashing intervention reported results by sex.^78^ Forest plots by intervention type, results of sensitivity analyses, and details on GRADE rating are provided in appendix 2 (pp 3–12, 14–16, 22–24).

## Discussion

In this systematic review and meta-analysis, we showed that WASH interventions reduced risk of diarrhoea. Drinking water interventions, specifically drinking water of higher quality and water filtered at POU, reduced risk of diarrhoea up to around 50%. Basic sanitation services without sewer connection resulted in a 21% reduction in diarrhoea risk and basic sanitation services with sewer connection resulted in a 47% reduction, compared with unimproved or limited sanitation. Combining water or sanitation interventions with other WASH interventions did not substantially increase reduction in diarrhoea. Handwashing promotion with or without broader hygiene education reduced diarrhoea by 30%. Only five (4%) of 124 included studies reported results by sex, showing the extent to which reporting of sex-disaggregated data is specifically limited in this context.

We found no additional reduction in disease from supplying piped drinking water on premises, compared with other improved sources. This finding could be due to two possible explanations. First, piped water in resource-constrained settings might be at high risk of faecal contamination because of inadequate or no disinfection; microbial growth and biofilms; leaks compounded by low pressure and intermittency, leading to backflow or groundwater intrusion; and intentional breaking of pipes to gain water access.^79^, ^80^ Second, intermittent piped water is a considerable issue in LMICs that affects both water quality and quantity, with hundreds of millions of people estimated to be affected.^80^ Studies in Ethiopia and Egypt have provided evidence for a higher risk of diarrhoea among individuals with an intermittent piped water supply than among those with a continuous piped supply.^81^, ^82^ Third, and related to the previous hypothesis, storage of drinking water is associated with considerable risk of recontamination^83^ and most of the piped water interventions on premises included in this analysis reported continuing household water storage.^35^, ^84^, ^85^, ^86^, ^87^, ^88^, ^89^

Other research suggests that providing water on premises is important for overall wellbeing^90^ through benefits to quality of life, such as the ability to reallocate time previously used for water collection to income generation or leisure,^91^ positive health effects (eg, on musculoskeletal disorders),^92^ reduced labour and calorie expenditure,^93^ and a potential increase in water quantity for other activities. These benefits might be particularly relevant for women who bear most of the burden of water collection globally.^34^, ^94^

We identified only two studies for the transition from improved water source on premises to improved source, on premises, and higher water quality,^24^, ^95^ and one study for the transition from improved water source on premises to improved source, on premises, and continuous supply.^85^ In these interventions, drinking water was not free from microbial contamination^24^, ^95^ and was still stored at household level^85^ after intervention implementation. The two transitions to improved water source, on premises, and higher water quality and improved source, on premises, and continuous supply (figure 1) would ideally be combined into one transition that could more adequately represent safely managed drinking water services, as defined by the Joint Monitoring Programme for Water Supply, Sanitation and Hygiene. However, such a low-risk exposure level is currently not achieved in any study of drinking water in LMICs. Therefore, our largest estimated reduction in risk of diarrhoea from improvements to drinking water is likely to be an underestimate of what could be achieved by supplying safe and adequate drinking water.

Basic sanitation services without sewer connection were associated with a moderate reduction in risk of diarrhoea. Interventions providing basic sanitation services might not be able to sufficiently reduce faecal environmental contamination.^96^, ^97^, ^98^, ^99^ When considering basic sanitation, there was no evidence that risk of diarrhoea was associated with the level of community coverage (appendix 2 p 13). This finding could be due to how basic sanitation interventions often fail to adequately isolate excreta from the environment and due to human exposures at different stages along the sanitation chain, from containment to emptying, conveyance, treatment, and disposal or reuse.^100^ In addition, basic sanitation services have shown low levels of actual use.^96^, ^101^

This meta-analysis suggests that sewered sanitation had a greater effect on health than did onsite sanitation; however, there are several explanations for this finding, such as increased community coverage for sewered interventions and intervention setting. The contexts in which sewered or onsite sanitation are the appropriate options and, thus, the settings of the intervention studies, are likely to differ (eg, population density or disease burden). Furthermore, much of sewered sanitation worldwide is not connected to adequate treatment of waste water, which results in high environmental contamination with faeces and subsequently increased risks of diarrhoeal disease in adjacent communities.^102^, ^103^ However, the epidemiological studies identified in this systematic review did not examine the effects on downstream communities. In LMICs, the sanitary protection and effective treatment provided by both onsite and sewered sanitation systems are often of poor quality. There are major data gaps globally on the safe management of onsite and sewered sanitation services, including effective containment, faecal sludge management,^9^ and treatment of waste water, and we did not identify corresponding intervention studies. It is possible that greater effects on health could be achieved if excreta were safely managed along the full sanitation chain.

The pooled estimate for reduction in diarrhoea risk from handwashing interventions was based on interventions conducted at household level or community level in LMICs, as well as day-care institutions and primary schools in low-income, middle-income, and high-income settings. Around half of the handwashing interventions focused on handwashing promotion with or without soap provision, whereas the other half of interventions involved broader hygiene education, such as on food and kitchen hygiene or on safe drinking water and sanitation. Nevertheless, pooled RRs were remarkably close comparing household or community versus institutional setting, low-income, and middle-income versus high-income settings, or different hygiene interventions. A 30% reduction in the risk of diarrhoeal disease is impressive, given the known limitations of adherence to handwashing programmes. Given our reliance on interventions with suboptimal adherence, improvement in the fidelity of handwashing interventions would probably yield a greater impact on health.

This systematic review and meta-analysis has several limitations. We did not systematically search the grey literature, so we could have missed studies that were not published in peer-reviewed journals. Additionally, there was a potential for exposure misclassification, low compliance and response bias, and high heterogeneity across studies, as well as limitations related to the analysis at study level. Most of these limitations are reflected in the risk of bias and GRADE assessments and are also discussed in more detail in the previous publications.^5^, ^18^ The choice of WASH components and intervention approaches often depend on the study context (eg, population density, existing coverage, and geography); therefore, direct comparison between WASH interventions and even within improvements along the WASH service ladders might be confounded.

We rated the body of evidence for WASH interventions between moderate and very low certainty evidence (appendix 2 pp 22–24), mainly because of non-randomised study designs, risk of bias, and imprecision. Of note, the GRADE approach was developed for rating the evidence of health-care interventions.^29^ Public health interventions, such as WASH, are often complex and contain several interacting components, require multiple behaviours and target groups, and are often inherently difficult to randomise or mask, which makes them more likely to be rated as low or very low certainty evidence compared with interventions that can be better controlled, such as clinical interventions.^104^, ^105^

Results of the preceding WASH review^5^ are largely consistent with the findings in this meta-analysis; however, this review found lower reductions in risk of diarrhoea with drinking water of higher quality supplied on premises. Scarce evidence was available for this exposure category; thus, addition of new evidence substantially changed estimates. A previous Cochrane review on drinking water interventions, which was last updated in 2014 and had narrower inclusion criteria than in this review, found similar RRs for water treatment at POU but did not provide pooled estimates for source-based water improvements, piped water to households, or higher water quality supplied on premises.^1^ Freeman and colleagues^3^ found an overall RR of 0·77 (95% CI 0·66–0·91) for the effect of sanitation interventions on risk of diarrhoea in children, which is close to our pooled estimate of all sanitation interventions. A systematic review looking specifically at the association between sewer connection and risk of diarrhoea found an overall RR of 0·70 (0·61–0·79) for all sewer studies and of 0·41 (0·27–0·61) when sanitation service level at baseline or in the comparison group was very poor.^14^ A Cochrane review on handwashing promotion and risk of diarrhoea found similar point estimates for the effects of handwashing promotion on reduction in risk of diarrhoea in communities across LMICs (0·71 [0·62–0·81]) and in day-care facilities and schools in high-income countries (0·70 [0·58–0·85]).^2^ By contrast, estimates used by the Global Burden of Diseases, Injuries, and Risk Factors Study (GBD) 2019^10^ differed from the findings of this systematic review and meta-analysis. GBD 2019 estimated substantially larger reductions in risk of diarrhoea with piped water compared with unimproved water (0·64 [0·56–0·73]), high-quality piped water compared with unimproved water (0·20 [0·09–0·49]), and compared with unimproved untreated water (0·09 [0·04–0·23]), and sewered sanitation or septic tanks compared with unimproved sanitation (0·31 [0·28–0·34]; appendix 2 pp 24–27).^106^

Our findings suggest that movement up the water, sanitation, and hygiene ladders confers increased protection from diarrhoeal pathogens. This meta-analysis presents effect estimates by intervention type and by the respective comparison WASH exposure level, instead of combining results of heterogeneous water, sanitation, and hygiene interventions, and different comparison exposure levels. We believe that additional evidence on the provision of basic WASH services, such as POU water treatment, provision of basic sanitation services, and hygiene promotion, is unlikely to change the results of this analysis to a major extent. This assumption is supported by the comparison with results from previous reviews. Although evidence on the effects of providing drinking water of higher quality supplied on premises and basic sanitation services with sewer connection on health is scarce, data on the effects of supplying safely managed WASH services in LMICs are completely missing. Provision of safe WASH services would include a combination of providing clean and continuously available piped water into homes; access to sanitation at home that conveniently, safely, and effectively disposes of faeces; and access to basic hygiene services, which are essential for different hygiene behaviours. Without evidence on these transitions, which would ideally be delivered together and consider further disease pathways (eg, targeting animal faeces and food hygiene), we are not completely capturing the health gains that could be achieved by the provision of safe and adequate WASH services across LMICs.

**This online publication has been corrected. The corrected version first appeared at thelancet.com on June 15, 2023**

## Data sharing

All data collected for this systematic review and meta-analysis, including search strategies, the review protocol, data extraction sheets, and analytical codes, are available immediately following publication without end date to anyone for any purpose and are either published in the appendices or can be accessed through the corresponding author.

**This online publication has been corrected. The corrected version first appeared at thelancet.com on July 21, 2022**

## Declaration of interests

JW and BG report grants from the UK Foreign, Commonwealth and Development Office, during the conduct of the study. SH reports grants from the National Institute of Environmental Health Sciences, during the conduct of the study. MB reports grants from Bill & Melinda Gates Foundation during the conduct of the study. BFA reports grants from National Institutes of Health, and grants and non-financial support from Bill & Melinda Gates Foundation, outside the submitted work. MCF reports personal fees from Reckitt, outside the submitted work. AM has received funding as a statistical consultant on *The Lancet* Commission on water, sanitation and hygiene, and health to analyse the associations between water, sanitation, hygiene, and handwashing and child health using Multiple Indicator Cluster Survey data and to complete an individual-participant data meta-analysis of WASH trials on pathogen contamination in the environment and child health. All other authors declare no competing interests.
