
JOURNAL INFORMATION
==============================
NLM Title Abbreviation: Lancet
Journal ID: Lancet
NLM Journal ID: 2985213R

Lancet (London, England)

ISSN: 0140-6736
EISSN: 1474-547X

ARTICLE INFORMATION
==============================
PMCID: PMC9343320
PMID: 35871811
DOI: 10.1016/S0140-6736(22)01341-1
Article ID: S0140-6736(22)01341-1
Article version: 1
Subjects: Department of Error

Department of Error

Print publication date: 2022 Jul 23
Volume: 400
Issue: 10348
First page: 272
Last page: 272
Copyright: .
Copyright year: 2022
Copyright holder: Elsevier Ltd
License: This is an open access article under the CC BY license (http://creativecommons.org/licenses/by/3.0/).
License URL: https://creativecommons.org/licenses/by/3.0/

Erratum for: Effectiveness of interventions to improve drinking water, sanitation, and handwashing with soap on risk of diarrhoeal disease in children in low-income and middle-income settings: a systematic review and meta-analysis 400 10345 2 7 2022 27 40 Lancet (London, England) 10.1016/S0140-6736(22)00937-0 PMC9251635 35780792

==============================
Wolf J, Hubbard S, Brauer M, et al. Effectiveness of interventions to improve drinking water, sanitation, and handwashing with soap on risk of diarrhoeal disease in children in low-income and middle-income settings: a systematic review and meta-analysis. Lancet 2022; 400: 48–59—In figure 2A of this Article, “Improved or limited sanitation” should have read “Unimproved or limited sanitation”. This correction has been made as of July 21, 2022.

