
JOURNAL INFORMATION
==============================
NLM Title Abbreviation: Lancet
Journal ID: Lancet
NLM Journal ID: 2985213R

Lancet (London, England)

ISSN: 0140-6736
EISSN: 1474-547X

ARTICLE INFORMATION
==============================
PMCID: PMC10401716
PMID: 37330745
DOI: 10.1016/S0140-6736(23)01199-6
Article ID: S0140-6736(23)01199-6
Article version: 1
Subjects: Department of Error

Department of Error

Print publication date: 2023 Jun 17
Volume: 401
Issue: 10393
First page: 2040
Last page: 2040
Copyright: .
Copyright year: 2023
Copyright holder: Elsevier Ltd
License: This is an open access article under the CC BY license (http://creativecommons.org/licenses/by/3.0/).
License URL: https://creativecommons.org/licenses/by/3.0/

Erratum for: Effectiveness of interventions to improve drinking water, sanitation, and handwashing with soap on risk of diarrhoeal disease in children in low-income and middle-income settings: a systematic review and meta-analysis 400 10345 2 7 2022 27 40 Lancet (London, England) 10.1016/S0140-6736(22)00937-0 PMC9251635 35780792

==============================
Wolf J, Hubbard S, Brauer M, et al. Effectiveness of interventions to improve drinking water, sanitation, and handwashing with soap on risk of diarrhoeal disease in children in low-income and middle-income settings: a systematic review and meta-analysis. Lancet 2022; 400: 48–59—In this Article, the first line of the Findings in the Summary should have read “21 290 records were identified from the search, of which 124 studies were included, providing 83 water (62 616 children), 20 sanitation (40 799 children), and 41 hygiene (98 416 children) comparisons.” The first line of the Results should have read “21 290 records were identified from the search, of which 15 634 were screened and 83 assessed for eligibility (figure 3).” Figure 3 has been updated. The appendix of this Article has also been corrected. These corrections have been made as of June 15, 2023.

