# Supplementary material for: Effectiveness of interventions to improve drinking water, sanitation, and handwashing with soap on risk of diarrhoeal disease in children in low-income and middle-income settings: a systematic review and meta-analysis
Source: Lancet. 2022 Jul 2;400(10345):27–40. doi: 10.1016/S0140-6736(22)00937-0 (PMC9251635; doi:10.1016/S0140-6736(22)00937-0)
Supplement: Supplementary appendix [file mmc2.pdf]

THE LANCET

Supplementary appendix 2

This appendix formed part of the original submission and has been peer reviewed. We post it as supplied by the authors.

Supplement to: Wolf J, Hubbard S, Brauer M, et al. Effectiveness of interventions to improve drinking water, sanitation, and handwashing with soap on risk of diarrhoeal disease in children in low-income and middle-income settings: a systematic review and meta-analysis. *Lancet* 2022; **400:** 48–59.

Supplementary material

Table of contents

1. Participants of the initial expert meeting “Consultancy on Assessment of the burden of disease attributable to water, sanitation and hygiene”, London School of Hygiene and Tropical Medicine, 8-9 April 2013 1

2. Comparison of methods between current and previous systematic review and meta-analysis 2

3. Multiple comparisons in single intervention study 3

4. Subgroup meta-analyses and forest plots by intervention type 3

5. Assessment of covariates 13

6. Sensitivity analyses and separate analyses 14

7. Funnel plots by intervention type 16

8. Drinking water and sanitation service levels according to the JMP (service ladders) 21

9. GRADE assessment 22

10. Comparison with other reviews 24

References 28

1. Participants of the initial expert meeting “Consultancy on Assessment of the burden of disease attributable to water, sanitation and hygiene”, London School of Hygiene and Tropical Medicine, 8-9 April 2013

- Prof. Jamie Bartram, Gillings School of Global Public Health, University of North Carolina at Chapel Hill, USA
- Prof. Sandy Cairncross, Department of Disease Control, London School of Hygiene and Tropical Medicine, UK
- Prof. Thomas Clasen, Department of Disease Control, London School of Hygiene and Tropical Medicine, UK
- Prof Jack Colford, School of Public Health, Division of Epidemiology, University of California, USA
- Mr Oliver Cumming, Department of Disease Control, London School of Hygiene and Tropical Medicine, UK
- Prof. Valerie Curtis, Department of Disease Control, London School of Hygiene and Tropical Medicine, UK
- Prof. Alan Dangour, Department of Population Health, London School of Hygiene and Tropical Medicine, UK
- Prof. Paul Hunter, Norwich Medical School, University of East Anglia, UK
- Dr Richard Johnston, Water Supply and Treatment, EAWAG, Switzerland
- Dr Lorna Fewtrell, Centre for Research into Environment and Health, Aberystwyth University, UK
- Mr Bruce Gordon, Water Sanitation and Hygiene, Department of Public Health and Environment, World Health Organization
- Ms Jennifer de France, Water Sanitation and Hygiene, Department of Public Health and Environment, World Health Organization
- Dr Annette Prüss-Üstün, Evidence and Policy for Environmental Health, Department of Public Health and Environment, World Health Organization
- Dr Sophie Bonjour, Evidence and Policy for Environmental Health, Department of Public Health and Environment, World Health Organization
- Dr Jennyfer Wolf, Evidence and Policy for Environmental Health, Department of Public Health and Environment, World Health Organization

2. Comparison of methods between current and previous systematic review and meta-analysis

Table S1.1: Differences in methods between current and previous [1] systematic review and meta-analysis (SRMA)

		Current SRMA	Previous SRMA
Systematic review			
	Eligible study designs	Studies of interventions (randomized and non-randomized)	Studies of interventions (randomized and non-randomized); additionally observational studies without a clearly specified intervention analyzing cross-sectional household survey data applying specific matching methods (such as propensity score matching)
	Data extraction	Extraction of sex-disaggregated outcome data	No extraction of disaggregated outcome data.
Analysis			
	Bias assessment	Modified risk of bias assessment of individual studies, modified GRADE approach for assessing the certainty of a body of evidence	Risk of bias assessment of individual studies
	Bias adjustment	No adjustment for non-blinding (see paragraph below)	Additional analysis that adjusted results from unblinded POU water and hygiene intervention studies
	Sanitation analysis framework	Explicitly separates basic sanitation connected to sewer from non-sewered sanitation	Main analysis grouped all improved sanitation together
	Water analysis framework	Exposure scenarios follow more closely JMP water ladders: "improved, on premises", "improved on premises, higher water quality", "improved, on premises, continuous supply"; no adjustment for safe water storage in meta-regression analysis but as a separate analysis within the pool of POU chlorine studies	Previous higher level exposure scenarios referring to "piped water on premises"

GRADE: Grading of Recommendations, Assessment, Development and Evaluation; POU: point of use water quality interventions including chlorine, solar and filter treatment

Adjustment for non-blinding

Previously we adjusted effect estimates of all non-blinded hygiene interventions and also non-blinded POU drinking water interventions [1,2]. Hygiene and POU interventions might be more prone to non-blinding bias compared to other WASH interventions because they are often more directly delivered to households and they are more obviously aimed at improving health. Most types of WASH interventions cannot be blinded to participants such as handwashing promotion or access to a certain drinking water source or sanitation facility. We decided against adjusting effect estimates from hygiene and POU interventions for potential bias from non-blinding in this review. Non-blinding is one potential reason for biased results which likely exaggerates the intervention effect on diarrhoea. There are however many reasons for underestimated effect estimates on health such as exposure misclassification, incomplete implementation, or low compliance. Furthermore, the available POU studies which blinded participants to intervention status all analysed chlorine interventions [3–6], which we believe to have inherent limitations that limit their suitability for estimating the effect from non-blinding in POU

studies generally. Chlorine treatment is known to be ineffective against common diarrhoeagenic pathogens such as Cryptosporidium and Guardia [7] and may in fact be difficult to blind effectively (due to taste and smell) [4,6]. In addition, results that are not adjusted for one selected bias lend themselves better for comparisons with other reviews. Different recent WASH studies used negative control outcomes or active control groups [8,9] which may estimate the size of non-blinding bias for the particular setting and are potentially able to reduce both bias from lack of blinding and study drop-out [10].

3. Multiple comparisons in single intervention study

We combined separate effect estimates across intervention arms that were reported in a single intervention study and that fell within the same category (e.g., filtering water at POU using different technologies). When multiple independent effect estimates matching our exposure scenarios were given within a single intervention study we included independent subgroups (e.g., separate intervention and separate control group). In the case of multiple comparisons within a single intervention study (e.g., effect estimates for different POU water interventions) but with the same control group or different effect sizes across relevant age groups or for the same individuals over time, effect estimates were combined using methods described in Borenstein et al. [11]. Effect estimates from different participants, e.g. from different relevant age groups, were combined as independent subgroups (e.g., [12]), whereas different effect estimates on the same participants, e.g., collected at different time points, were combined taking into account the correlation between the effect estimates (e.g., [13–15]). Multiple comparisons within a single intervention study without separate control groups were not combined if the groups were not sufficiently similar (e.g., water intervention separately and water intervention plus hygiene education). In these cases, including factorial designs, we derived a single pair-wise comparison of the most comprehensive intervention compared with the least comprehensive intervention. As an example: an intervention providing an improved water supply on premises would be regarded as more comprehensive compared to an intervention providing an improved off-premises water supply (see Figure 1 of the manuscript). When separate effect estimates were given for a water or sanitation intervention alone or in combination with another WASH component, we included the effect estimate for the water or sanitation intervention not combined with other WASH components (e.g., water interventions without an additional hygiene or sanitation component).

4. Subgroup meta-analyses and forest plots by intervention type

Drinking water

Table S2.2: Results of subgroup meta-analysis by water intervention types

	**Reference service level**	**Number of comparisons**	**Effect size (95% CI)**	**Transition (Fig 1)**	**GRADE**
**Intervention type**					
Improved, not on premises	Unimproved	10	0·76 (0·60, 0·95), I^2=98%	a	⊕◯◯◯
Improved, on premises	Unimproved (n=3), improved, not on premises (n=6)	9	0·87 (0·68, 1·12), I^2=85%	b[3]	⊕◯◯◯
Improved, on premises, higher water quality	Improved, on premises	2	0·55 (0·24, 1.27), I^2=76%	c	⊕◯◯◯
Improved, on premises, continuous supply	Improved, on premises	1	0·93 (0·83, 1·04)	d	⊕◯◯◯
POU, chlorine[1]	Unimproved (n=17), improved,	25	0·69 (0·60, 0·78), I^2=89%	e[3]	⊕⊕⊕◯

	not on premises (n=8)				
POU, solar	Unimproved (n=6), improved, not on premises (n=7)	13	0·69 (0·62, 0·77), I²=54%	f[3]	⊕⊕⊕◯
POU, filter[2]	Unimproved (n=15), improved, not on premises (n=8)	23	0·51 (0·41, 0·65), I²=80%	g[3]	⊕⊕⊕◯
Total		83	0·68 (0·63, 0·74), I²=92%		

POU: point-of-use; ⊕◯◯◯: very low certainty evidence; ⊕⊕◯◯: low certainty evidence; ⊕⊕⊕◯: moderate certainty evidence; ⊕⊕⊕⊕: high certainty evidence; [1] some POU chlorine interventions provide an additional safe storage container, [2] filter includes ceramic filter, biosand filters and other filter technologies, [3] if reference service level: improved, not on premises

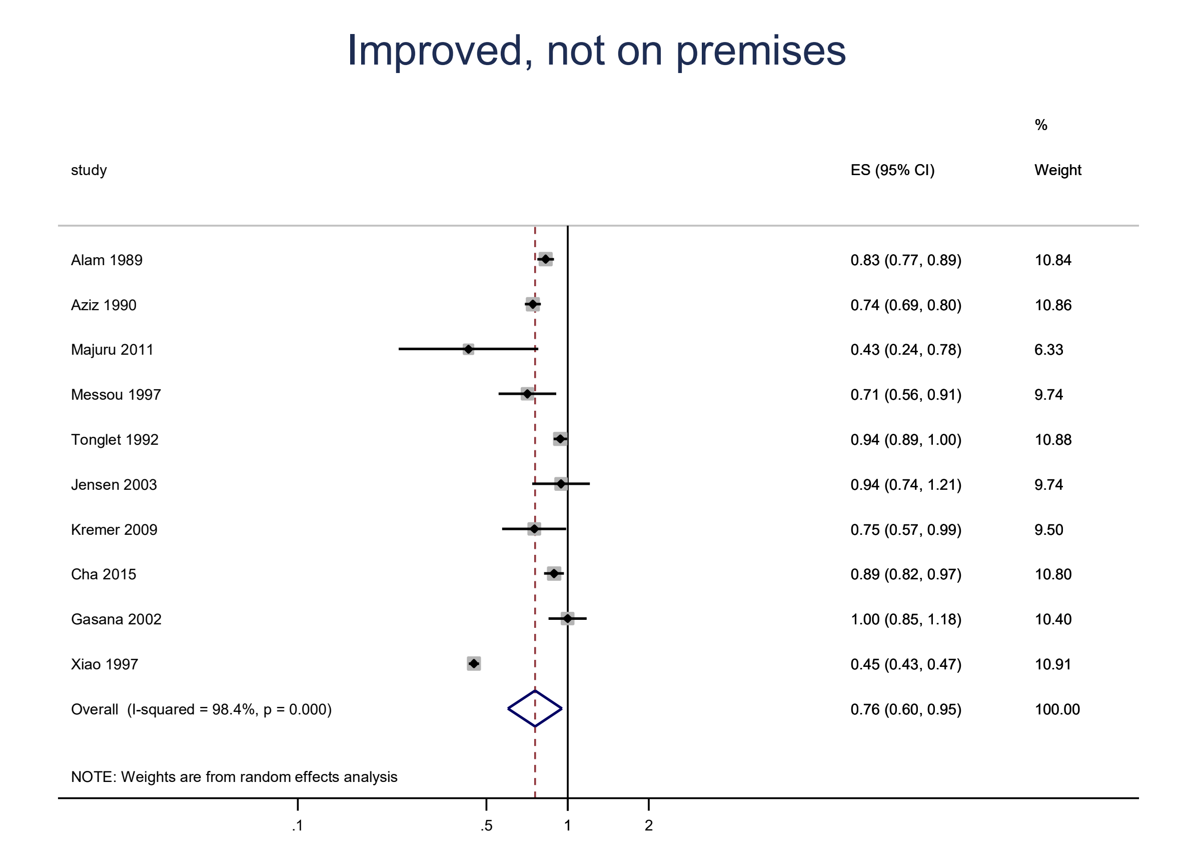

Figure S1.1: Forest plot of drinking water interventions: improved, not on premises

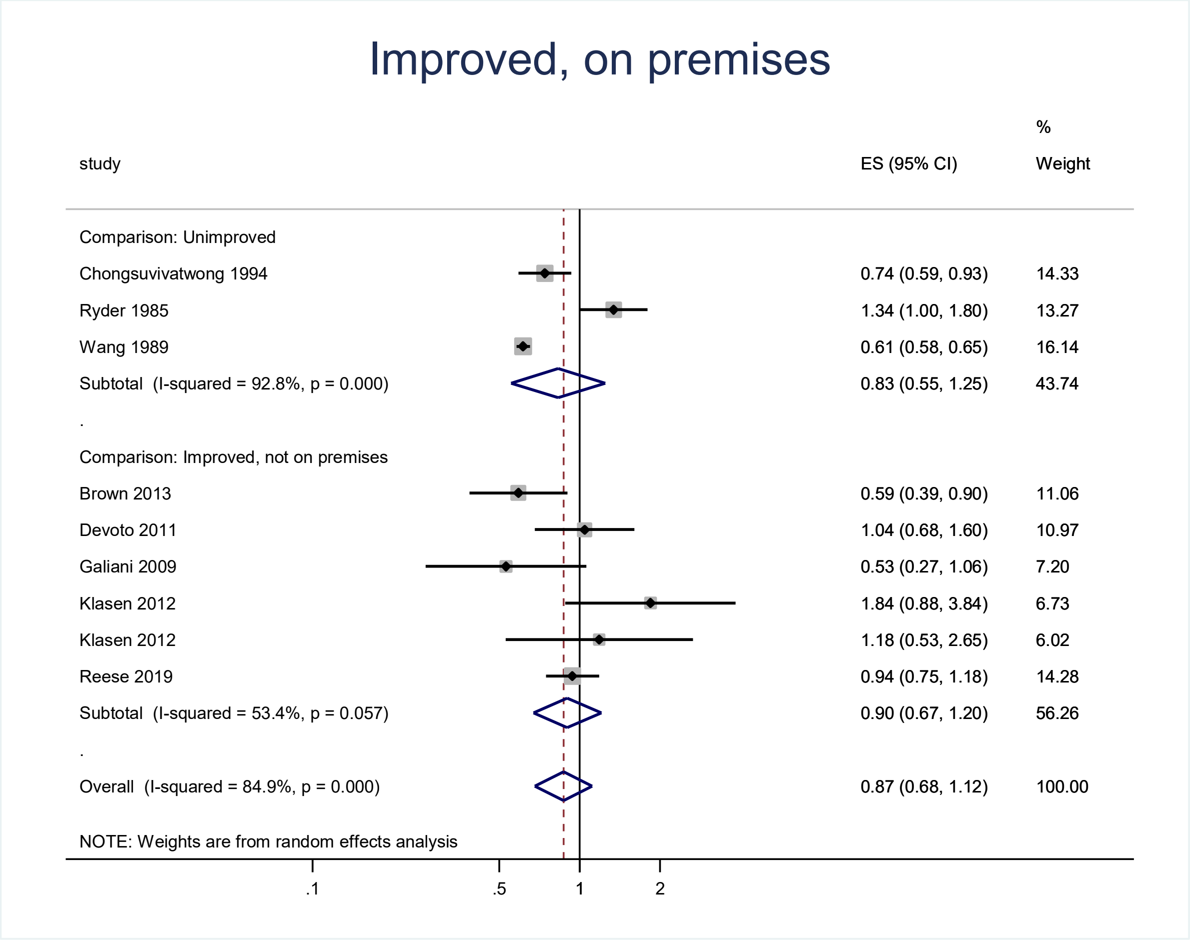

Figure S1.2: Forest plot of drinking water interventions: improved, on premises

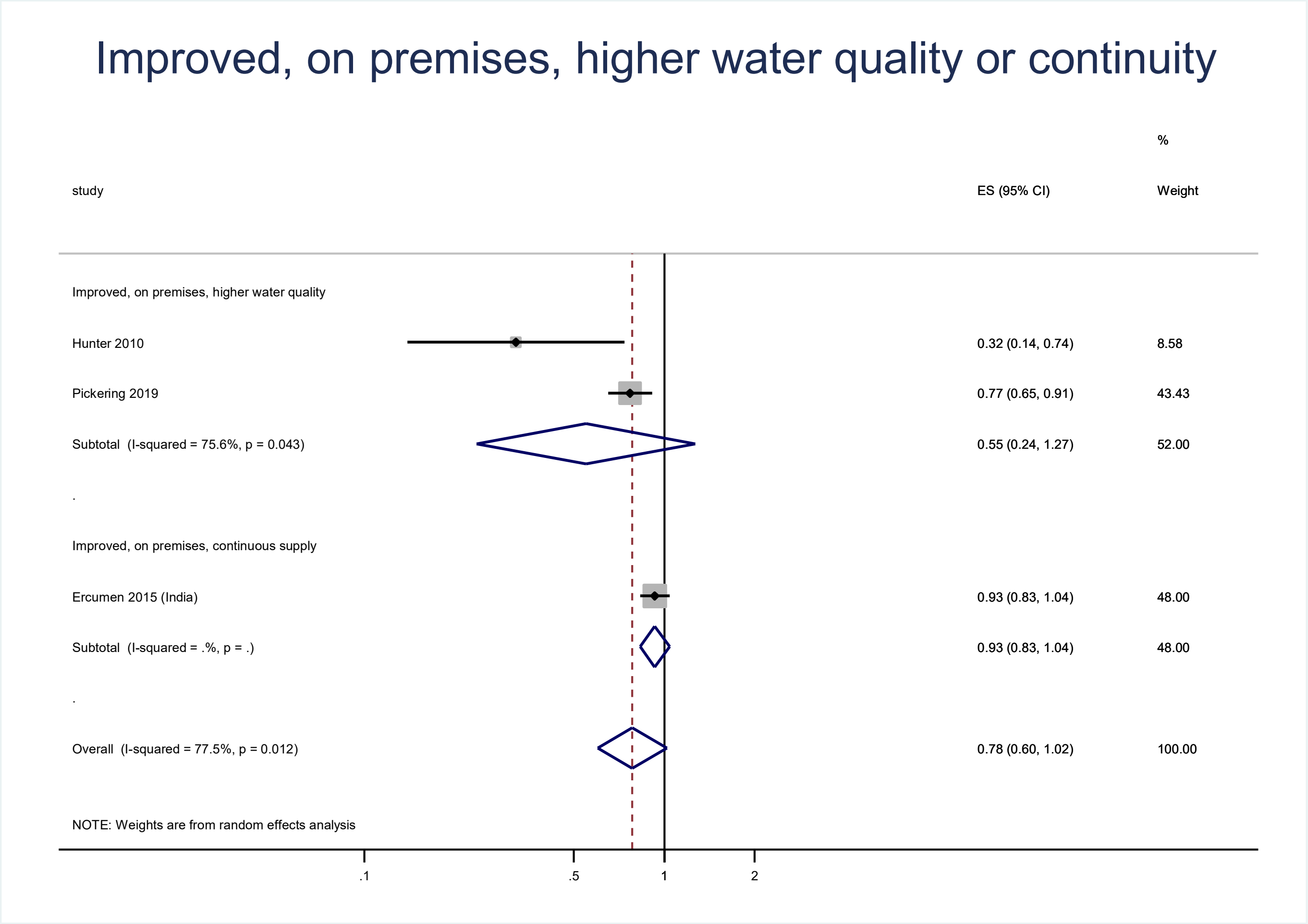

Figure S1.3: Forest plot of drinking water interventions: improved, on premises, higher water quality or continuity

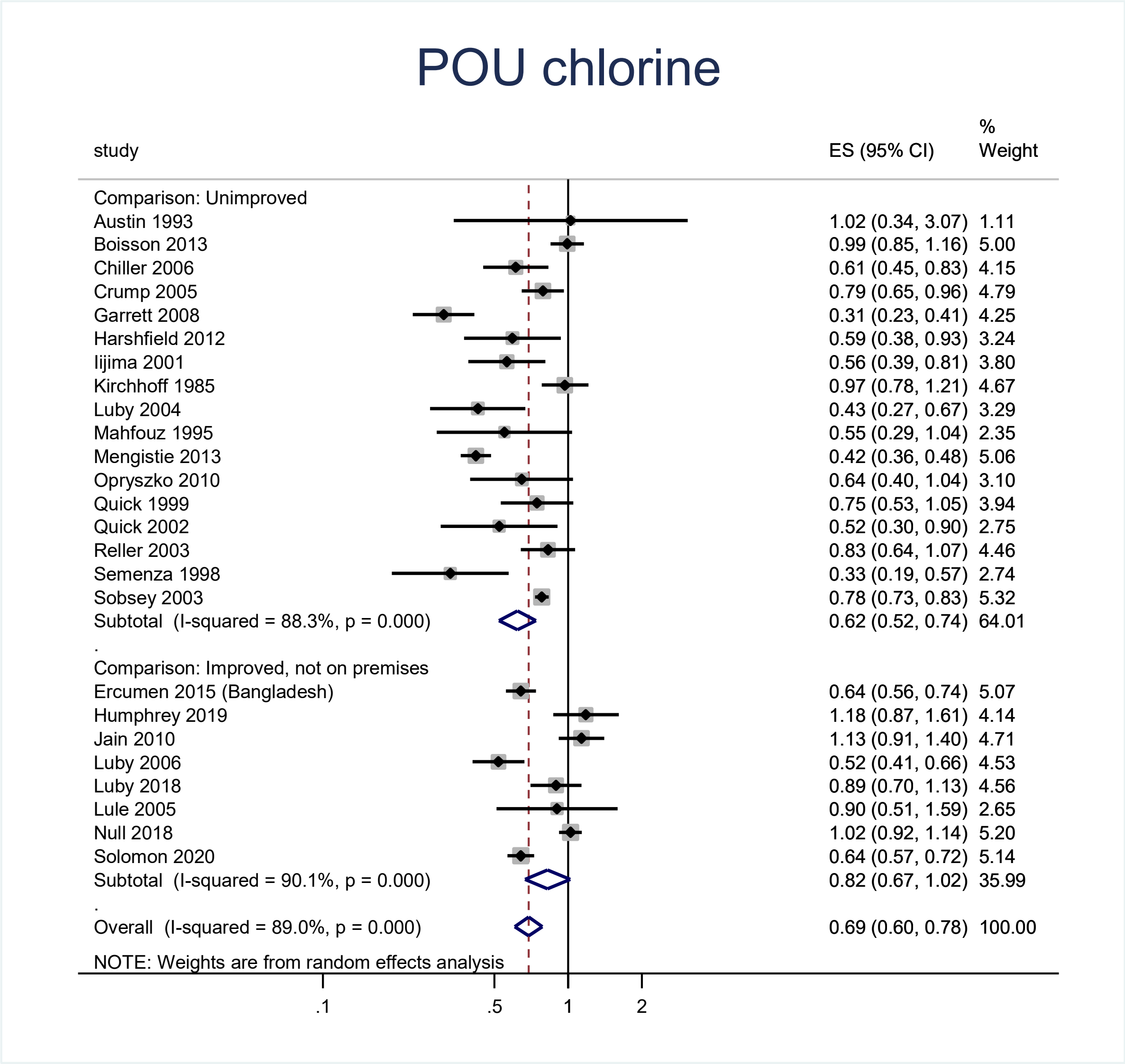

Figure S1.4: Forest plot of drinking water interventions: point-of-use (POU) chlorine treatment

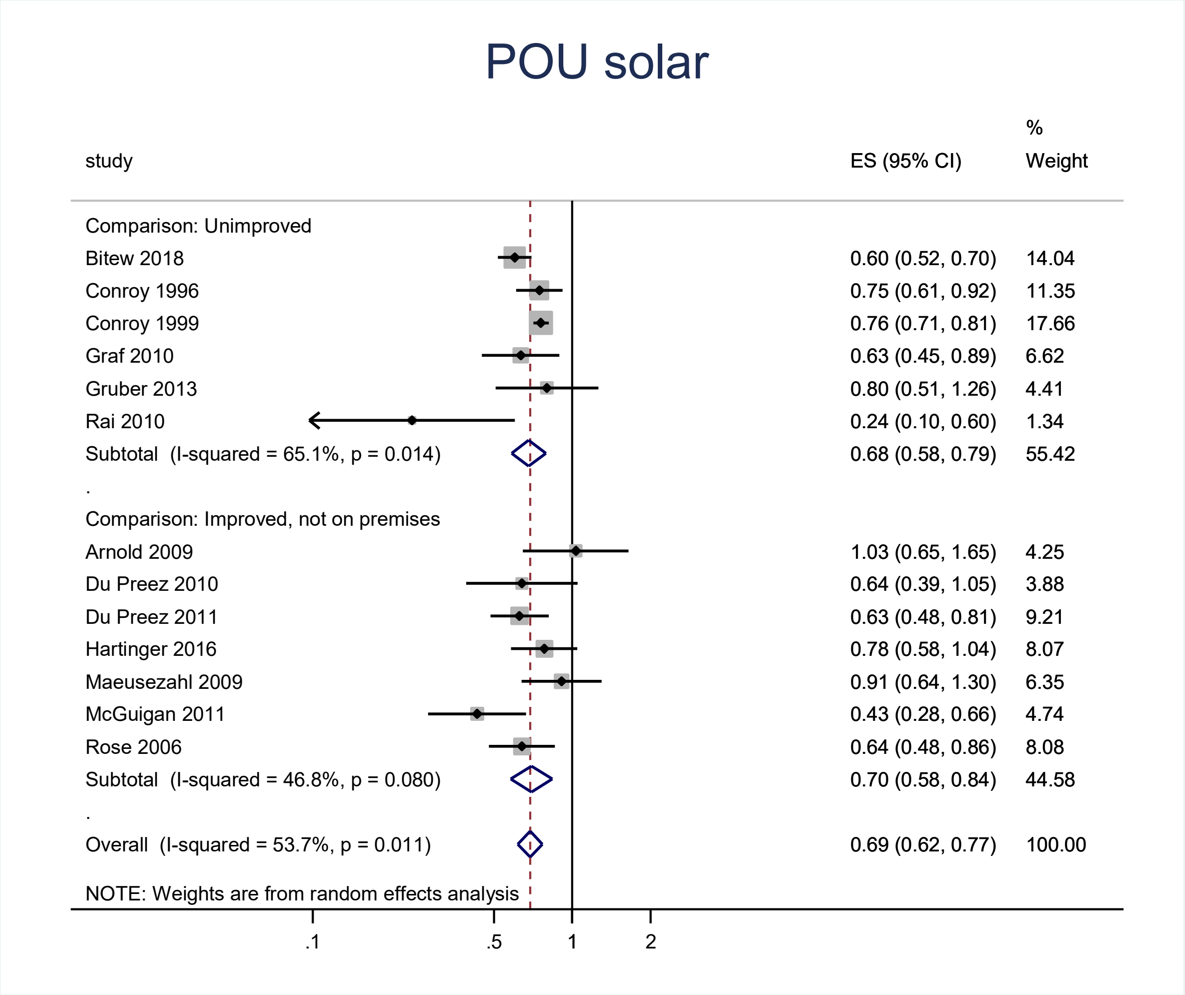

Figure S1.5: Forest plot of drinking water interventions: point-of-use (POU) solar treatment

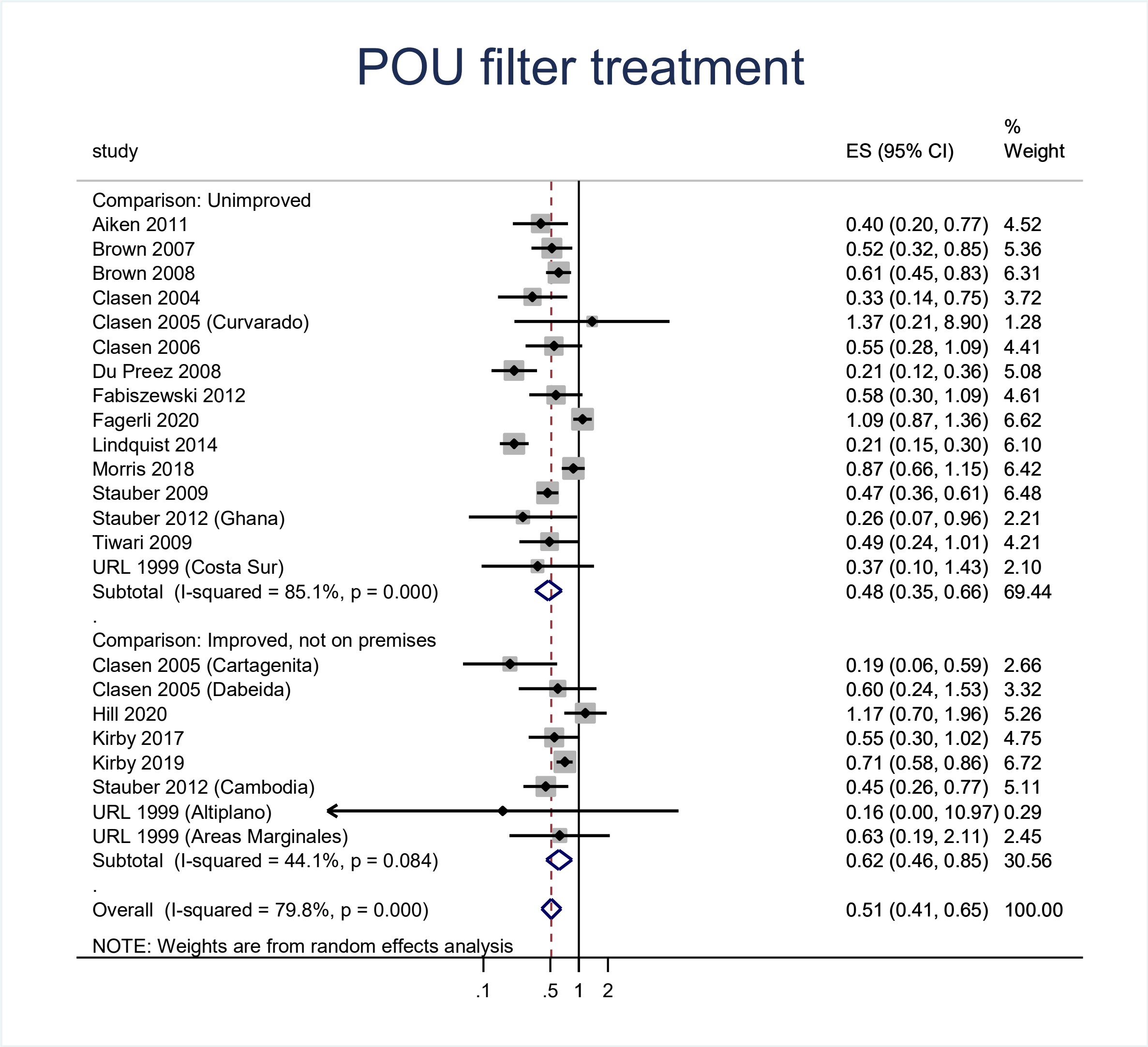

Figure S1.6: Forest plot of drinking water interventions: point-of-use (POU) filter treatment

Sanitation

Table S3.3: Results of subgroup meta-analysis by sanitation intervention types

	Reference service level	Number of comparisons	Effect size (95% CI)	Transition (Fig 2)	GRADE
**Intervention type**					
Basic sanitation services, without sewer connection	Unimproved/limited sanitation	15	0·82 (0·71, 0·94), I^2=86%	a	⊕⊕⊕◯
Basic sanitation services, with sewer connection	Unimproved/limited (n=2), basic sanitation services, without sewer connection (n=3)	5	0·63 (0·32, 1·26), I^2=94%	b[1]	⊕◯◯◯

⊕◯◯◯: very low certainty evidence; ⊕⊕◯◯: low certainty evidence; ⊕⊕⊕◯: moderate certainty evidence; ⊕⊕⊕⊕: high certainty evidence, [1] if reference service level: basic sanitation services, without sewer connection

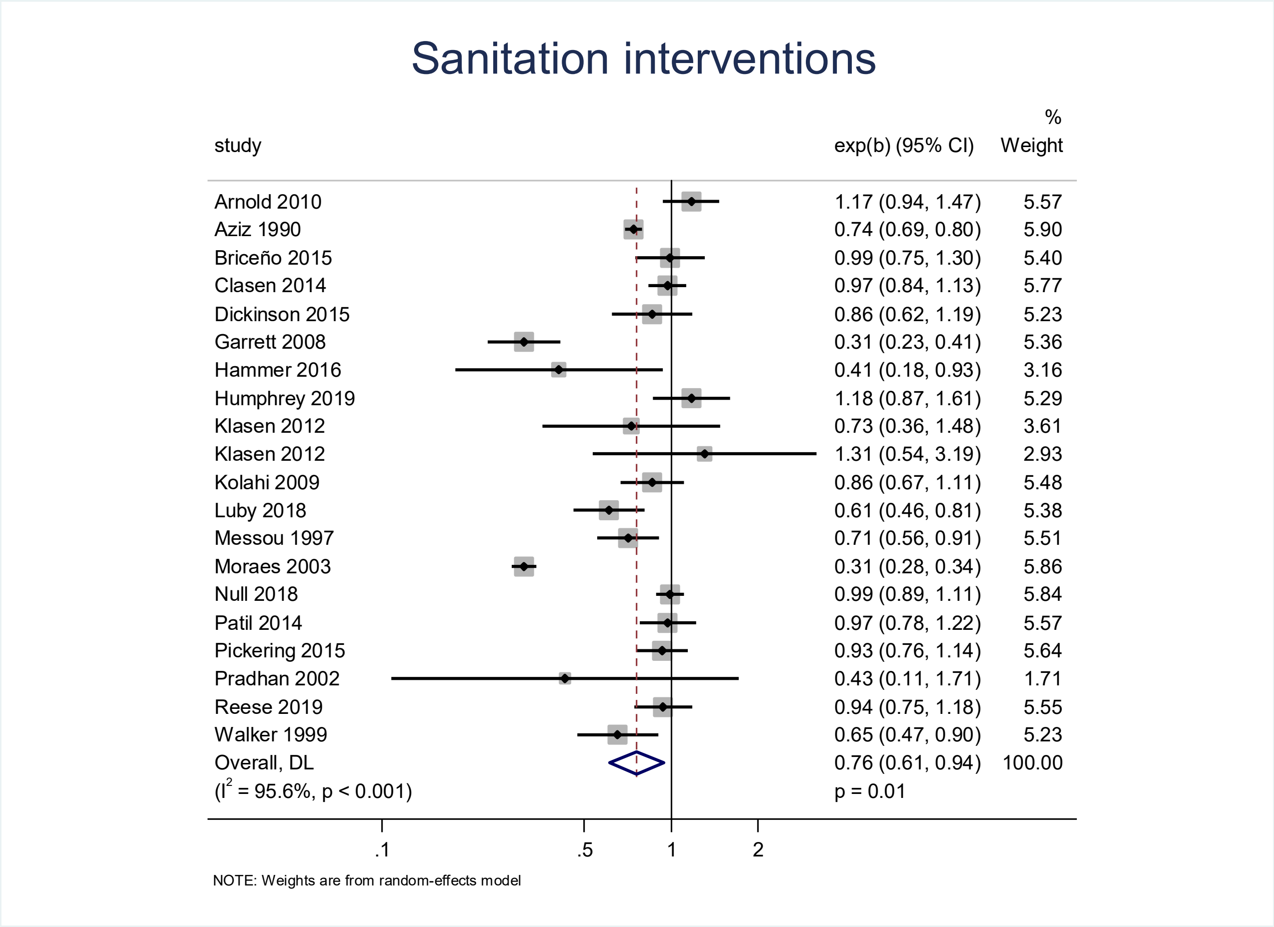

Figure S1.7: Forest plot of all sanitation interventions

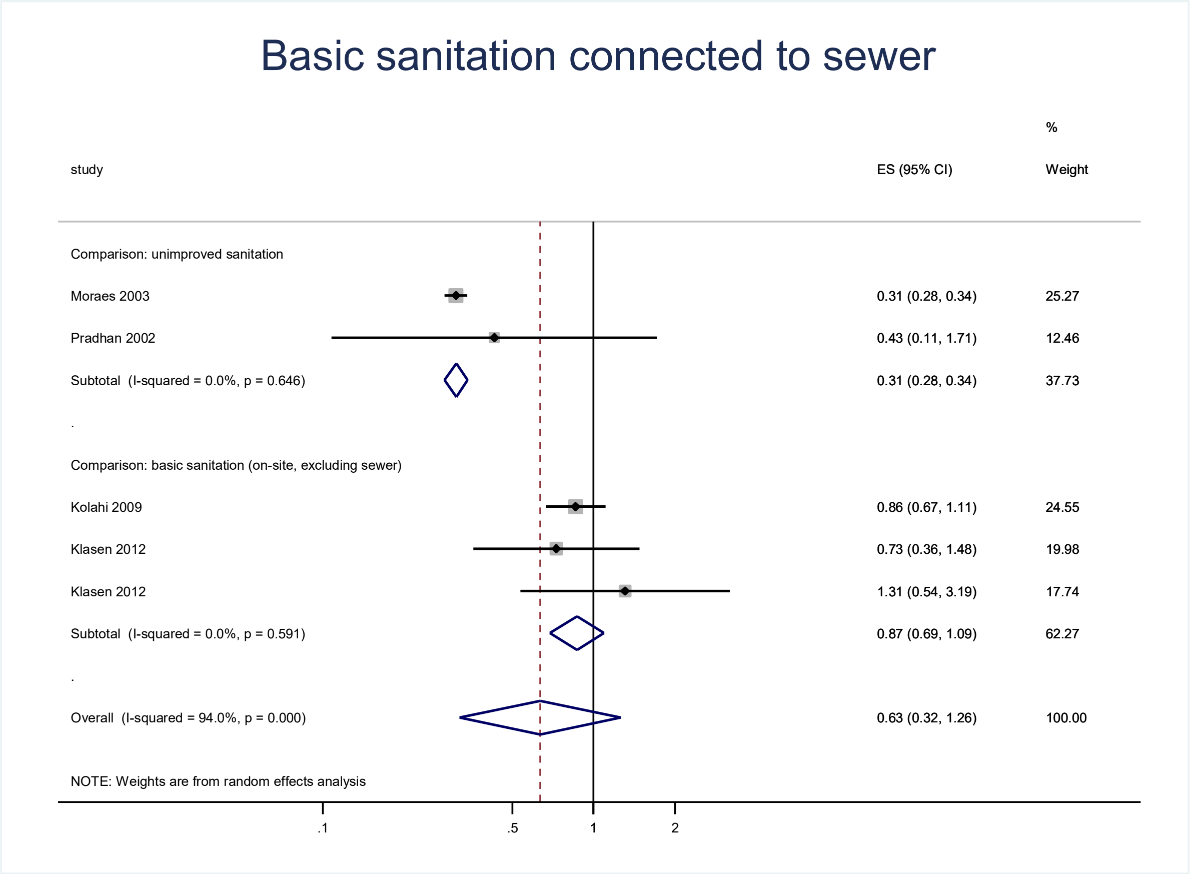

Figure S1.8: Forest plot of sanitation interventions: basic sanitation connected to sewer by comparison sanitation access

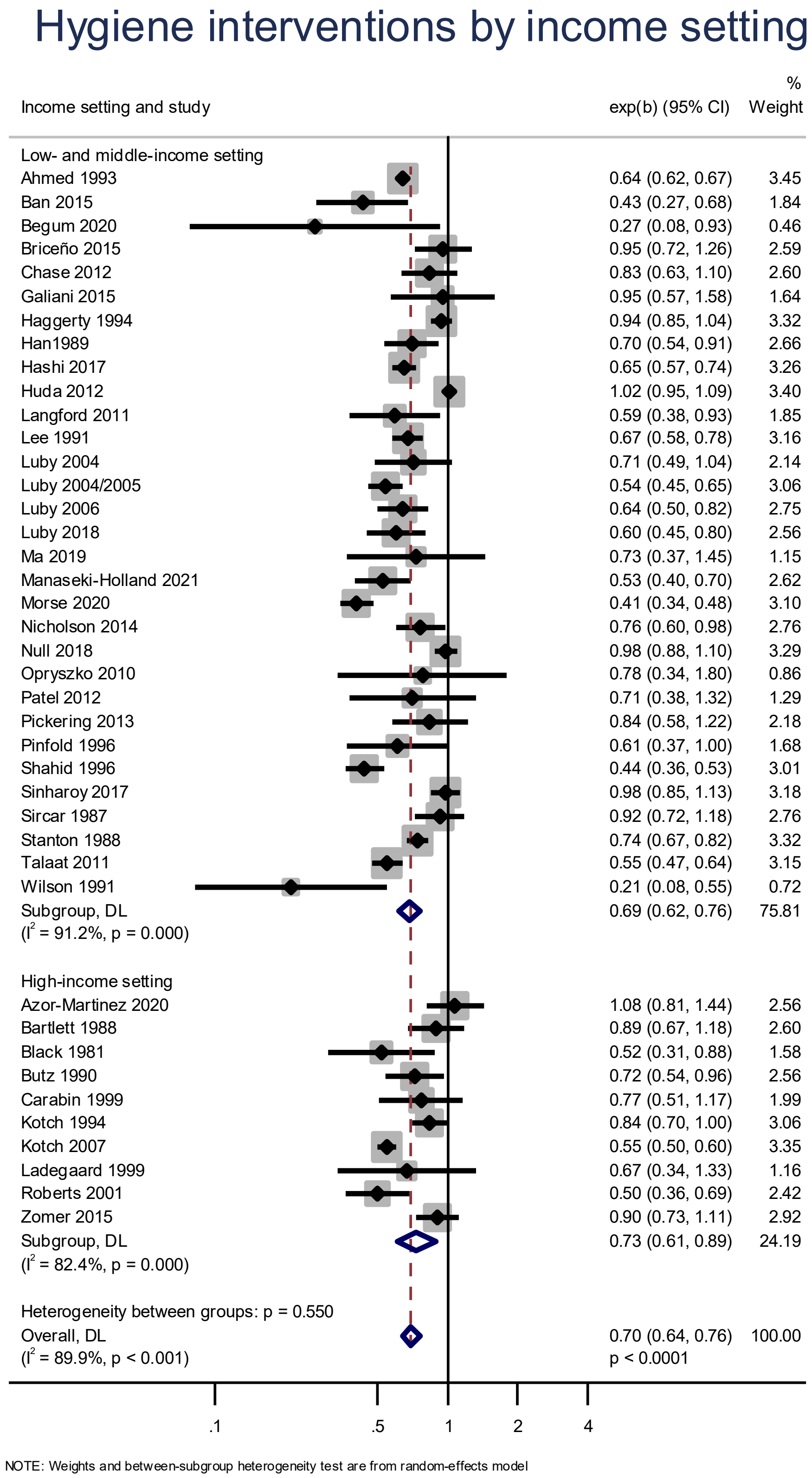

Figure S1.9: Forest plot of hygiene interventions by income setting

5. Assessment of covariates

Analysis of drinking water interventions

Covariates were examined one-by-one in the meta-regression model containing the seven binary variables indicating transitions between exposure scenarios (Figure 1 in manuscript) and a binary covariate for combined intervention.

Table S1.4: Covariates examined in the analysis of drinking water interventions

Covariate	Reference level	type	Effect estimate (95% CI)	p-value	Missing values
Improved sanitation	Unimproved sanitation	Binary	0·90 (0·72, 1·13)	0·35	2
Rural setting	Urban/periurban or mixed setting	Binary	1·08 (0·91, 1·27)	0·37	0
Time of follow-up ≥12 months*	Time of follow-up <12 months	Binary	1·12 (0·96, 1·31)	0·14	0
Time of follow-up by one month increase	NA	Discrete (one month-steps)	1·00 (1·00, 1·01)	0·28	0
Randomized	Non-randomized/ quasi-randomized	Binary	1·08 (0·86, 1·36)	0·48	0

*median duration of drinking water interventions: 11 months

Analysis of sanitation interventions

Covariates were examined one-by-one in the meta-regression model containing the two binary variables indicating transitions between exposure scenarios (Figure 2) plus one pre-specified covariate combined intervention.

Table S1·5: Covariates examined in the analysis of sanitation interventions

Covariate	Reference level	type	Effect estimate (95% CI)	p-value	Missing values
Improved water access	Unimproved water access	binary	1·38 (0·94, 2·03)	0·10	1
Coverage with intervention sanitation facility ≥75% in the community	Coverage with intervention sanitation facility <75% in the community	Binary	0·89 (0·55, 1·42)	0·60	0
Sanitation promotion without provision of “hardware” (toilets/latrines or material for their construction)	Provision of sanitation “hardware”	Binary	0·91 (0·56, 1·47)	0·68	0
Time of follow-up ≥24 months*	Time of follow-up <24 months	Binary	1·60 (1·18, 2·17)	0·005	0
Time of follow-up by one month increase	NA	Discrete (one month-steps)	1·01 (1·00, 1·02)	0·03	0

Randomized	Non-randomized/ quasi-randomized	Binary	1·61 (0·98, 2·67)	0·06	0

* median duration of sanitation interventions: 24 months

Analysis of hygiene interventions including handwashing promotion

Covariates were examined one-by-one in the meta-regression model containing no other covariates.

Table S1·6: Covariates examined in the analysis of hygiene interventions

Covariate	Reference level	type	Effect estimate (95% CI)	p-value	Missing values
Handwashing with soap promotion exclusively	Broader hygiene education	Binary	1·04 (0·87, 1·25)	0·66	0
Soap provided	Soap not provided	Binary	0·93 (0·77, 1·12)	0·43	0
High-income setting	Low- and middle-income setting	Binary	1·07 (0·86, 1·32)	0·54	0
Community setting	Child care/ school setting	Binary	1·00 (0·82, 1·22)	0·99	0
Time of follow up ≥12 months*	Time of follow-up <12 months	Binary	1·08 (0·89, 1·30)	0·43	0
Time of follow-up by one month increase	NA	Discrete (one month-steps)	1·01 (1·0, 1·02)	0·26	0
Randomized	Non-randomized/ quasi-randomized	Binary	1·15 (0·91, 1·45)	0·25	0

* median duration of hygiene interventions: 9 months

6. Sensitivity analyses and separate analyses

Sensitivity analyses

Removing results from observations on all ages and children older than five years

As a sensitivity analysis, we excluded effect estimates on diarrhoea from studies reporting for all ages or children >5 years in meta-analyses of water (nine studies reported effect sizes for all ages and one study for children >5years), sanitation (one study for all ages) and hygiene (three studies for children >5 years) studies.

Removing the ten drinking water studies that reported effect estimates for all ages (n=9) or children >5years (n=1) resulted in a pooled RR of 0·71 (0·67, 0·76) compared to 0·68 (0·63, 0·74) including all studies.

Removing one sanitation study that reported effect estimates for all ages resulted in a pooled RR of 0·77 (0·61, 0·96) compared to 0·76 (0·61, 0·94) including all intervention studies.

Removing three studies that reported effect estimates for children >5years resulted in a pooled RR of 0·70 (0·64, 0·77) compared to RR 0·70 (0·64, 0·76) including all intervention studies.

Including results from survey data analyses

For comparability with previous reviews [1,2], we included results from survey data analyses using certain matching techniques in which no intervention was delivered to the study population in a sensitivity analysis (those survey data analyses are listed separately in Supplementary File 4).

Meta-analysis of all comparisons from drinking water studies including survey data analyses (nine comparisons from six separate studies) yielded the same point estimate as the meta-analysis including only comparisons from intervention studies (RR 0·68 (0·63, 0·73) compared to 0·68 (0·63, 0·74)). Effect estimates for transitions as shown in the water exposure scenario (**Fehler! Verweisquelle konnte nicht gefunden werden.**) only changed marginally except for the transition from *improved, not on premises* to *improved, on premises* (from RR 0·97 (0·75, 1·25) for intervention studies only to RR 0·84 (0·68, 1·04) for intervention studies plus survey data analyses) and the transition from *improved, on premises* to *improved, on premises, continuous supply* (from RR 0·93 (0·50, 1·74) for intervention studies only to RR 0·83 (0·50, 1·40) for intervention studies plus survey data analyses).

Meta-analysis of all sanitation studies including survey data analyses (eleven comparisons from eight separate studies) yielded a pooled estimate of RR 0·80 (0·70, 0·91) compared to RR 0·76 (0·61, 0·94) for intervention studies only. The effect estimate from meta-regression changed from RR 0·79 (0·61, 1·03) to 0·83 (0·72, 0·96) for the transition from *unimproved/limited sanitation* to *basic sanitation services, without sewer connection* and from RR 0·66 (0·41, 1·07) to RR 0·75 (0·52, 1·07) for the transition from *basic sanitation services, without sewer connection* to *basic sanitation services, with sewer connection*.

Meta-analysis of all hygiene studies including one survey data analysis yielded a pooled estimate of RR 0·69 (0·63, 0·76) compared to RR 0·70 (0·64, 0·76) for intervention studies only.

Accounting for clustering in WASH interventions delivered at group-level

Many WASH interventions are delivered at group-level and some of those included in this review were analysed without taking account of resulting clustered data. This leads to incorrectly estimated standard errors and confidence intervals that are usually too narrow. Estimating the intracluster correlation coefficient (ICC) and related design effects which can be used for approximating correct standard errors [16] is challenging in complex intervention studies, especially when large clusters are randomized. Both the ICC and design effects depend on cluster size, number of measurements per person, the age range of the study group, between cluster variability and other factors such as occurrence of localized diarrhoea epidemics [17].

Sanitation studies not accounting for clustering were typically published before 2010 [13,15,18–22], while those that did were published in 2010 or later [9,23–33].

We did not attempt to correct standard errors of WASH studies that did not appropriately account for clustering but conducted different sensitivity analyses: We excluded all studies published before 2010 in the water, sanitation and hygiene meta-analysis.

Removing drinking water studies published before 2010 (n=44) in a pooled RR of 0·71 (0·64, 0·79) compared to 0·68 (0·63, 0·74) including all drinking water intervention studies.

Removing sanitation studies published before 2010 (n=7) resulted in a pooled RR of 0·95 (0·86, 1·04) compared to 0·76 (0·61, 0·94) including all sanitation intervention studies.

Removing hygiene studies published before 2010 (n=23) resulted in a pooled RR of 0·75 (0·65, 0·88) compared to RR 0·70 (0·64, 0·76) including all hygiene intervention studies.

In addition, we removed the two sanitation studies not taking account of clustering with the largest effect on diarrhoea [18,20]. Removing the two sanitation studies not taking account of clustering with the largest effect on diarrhoea [18,20] resulted in a pooled RR of 0·88 (0·79, 0·97).

Safe storage in POU chlorine and filter intervention studies:

About half of the included chlorine interventions also provided a separate safe water storage container, i.e., a container with a narrow opening that prevents the introduction of objects. We examined all POU chlorine interventions in a separate meta-regression for an association between the provision of safe water storage and diarrhoeal disease reduction.

POU chlorine interventions that provided an additional safe storage container were associated with a RR for diarrhoea of 0·63 (0·51, 0·79) compared to a RR of 0·83 (0·68, 1·02) for POU chlorine interventions that did not (adjusted for combined intervention).

Some POU filter interventions include an intrinsic safe water storage component, such as many ceramic filters, while others do not. Providing safe water storage either as an inherent part of the intervention filter or provided as an additional storage container was not associated with the risk for diarrhoea in POU filter interventions (RR 0·92 (0·51, 1·66)).

POU water treatment by reference water level (unimproved or improved)

A total of 61 POU interventions are included. Of those, 38 compare POU treatment against a reference of unimproved and 23 against a reference of improved drinking water. This analysis suggests that the impact of POU intervention studies differs by the reference level of drinking water service: We find a RR of 0·58 (0·51, 0·66) for POU interventions conducted on or compared against unimproved and a RR of 0·73 (0·62, 0·86) for POU interventions conducted on or compared against improved water sources.

POU interventions providing sex-disaggregated data

For all four POU studies with sex-disaggregated data, reductions in diarrhoea were demonstrated for both males and females receiving the intervention compared to controls. For the POU filter studies, effects were greater for females in two studies [34,35] and greater for males in the third [36]. There were no significant differences in the proportion of males and females with diarrhoea in the cross-sectional chlorine study [37].

7. Funnel plots by intervention type

Drinking water

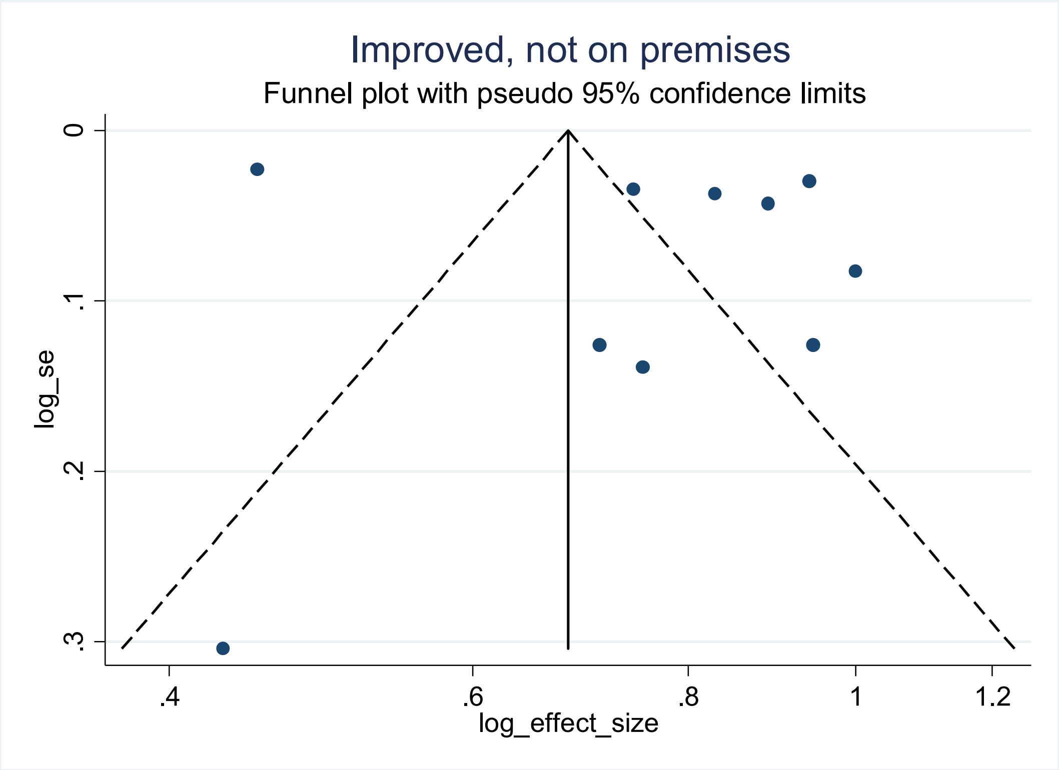

Figure S1·10: Funnel plot of drinking water interventions: improved, not on premises

Egger test: p = 0·36

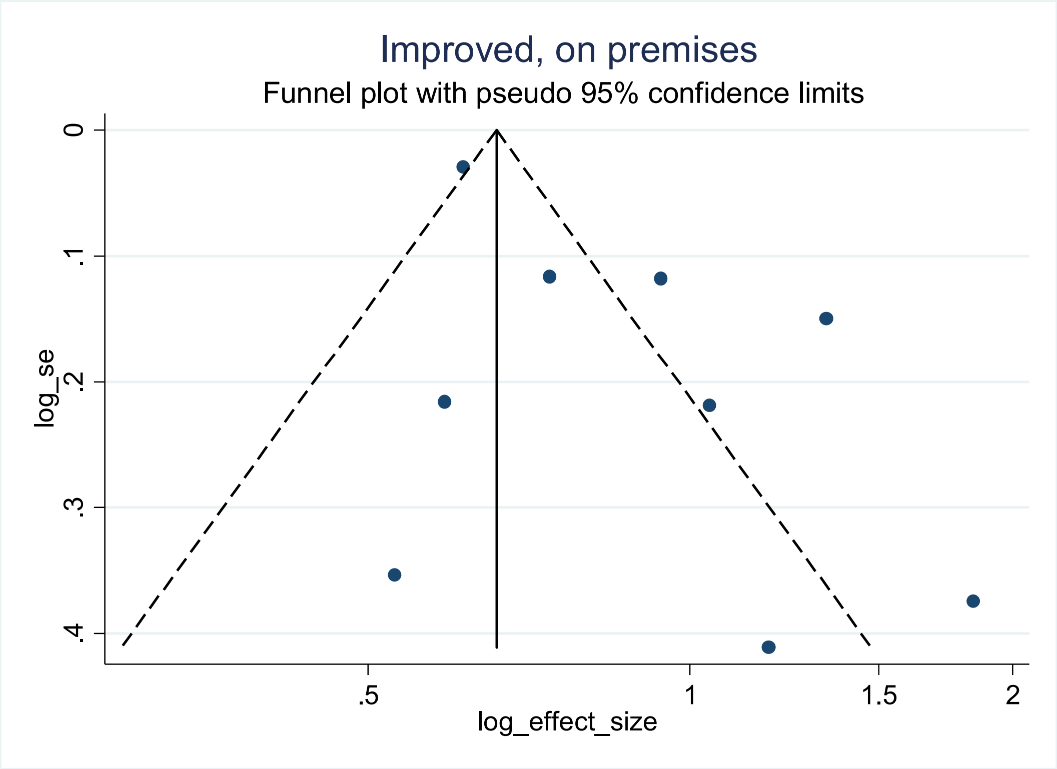

Figure S1·11: Funnel plot of drinking water interventions: improved, on premises

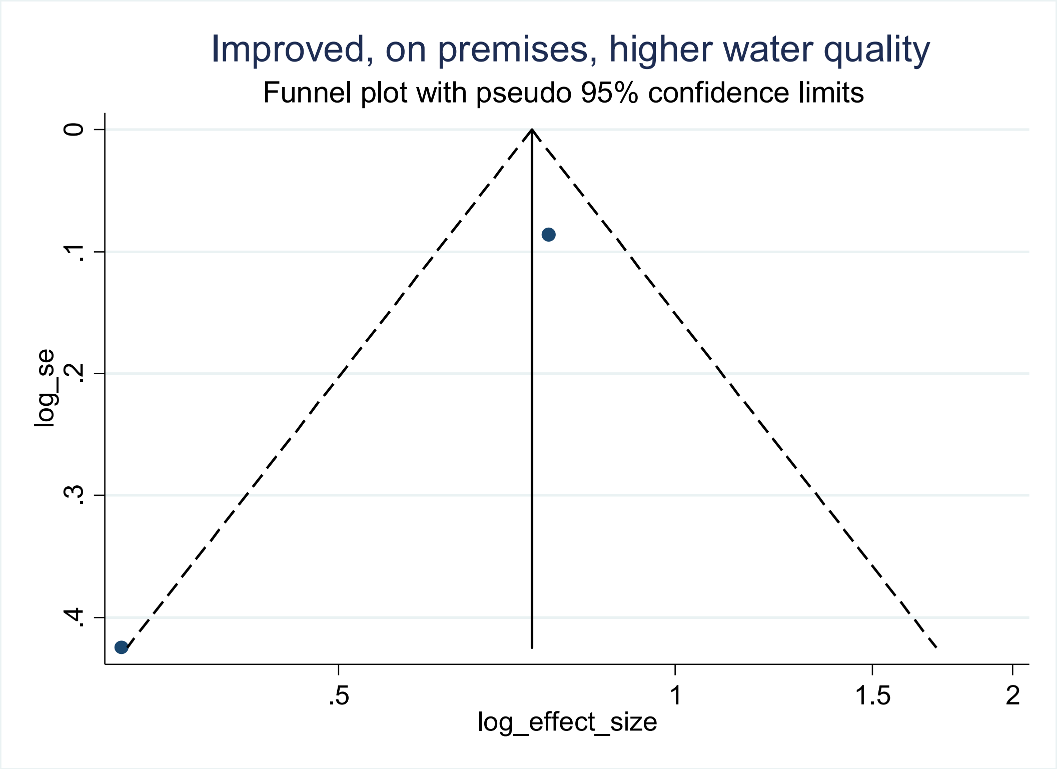

Figure S1·12: Funnel plot of drinking water interventions: improved, on premises, higher water quality

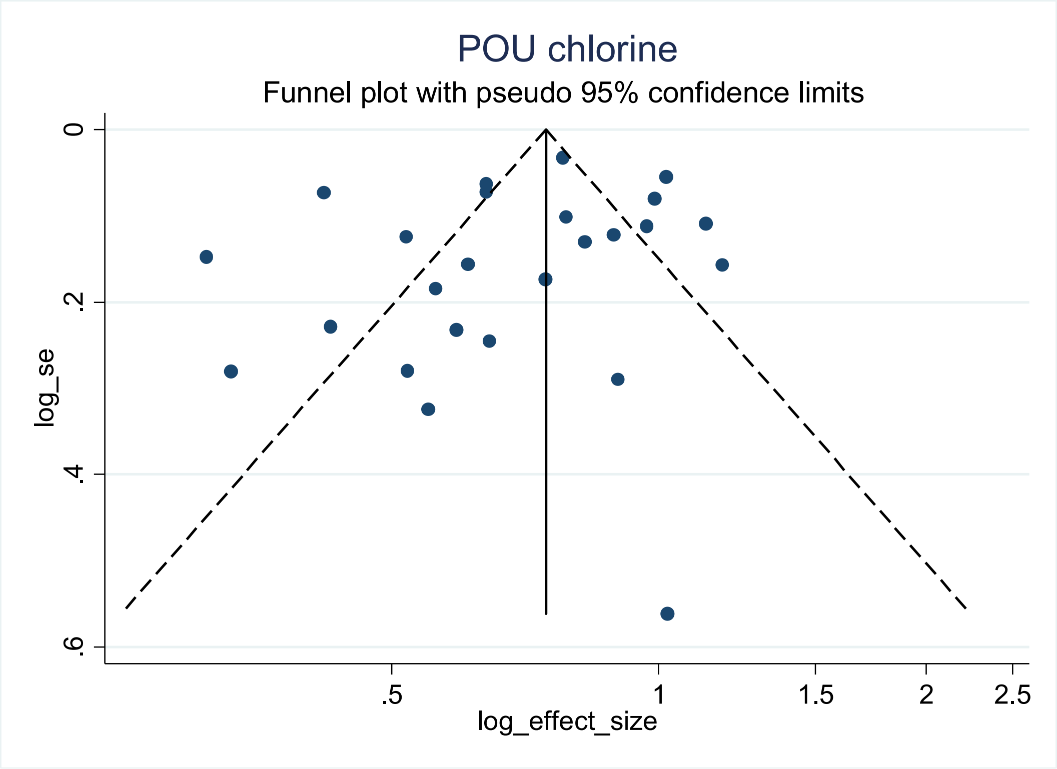

Figure S1·13: Funnel plot of drinking water interventions: point-of-use (POU) chlorine treatment

Egger test: p = 0·28

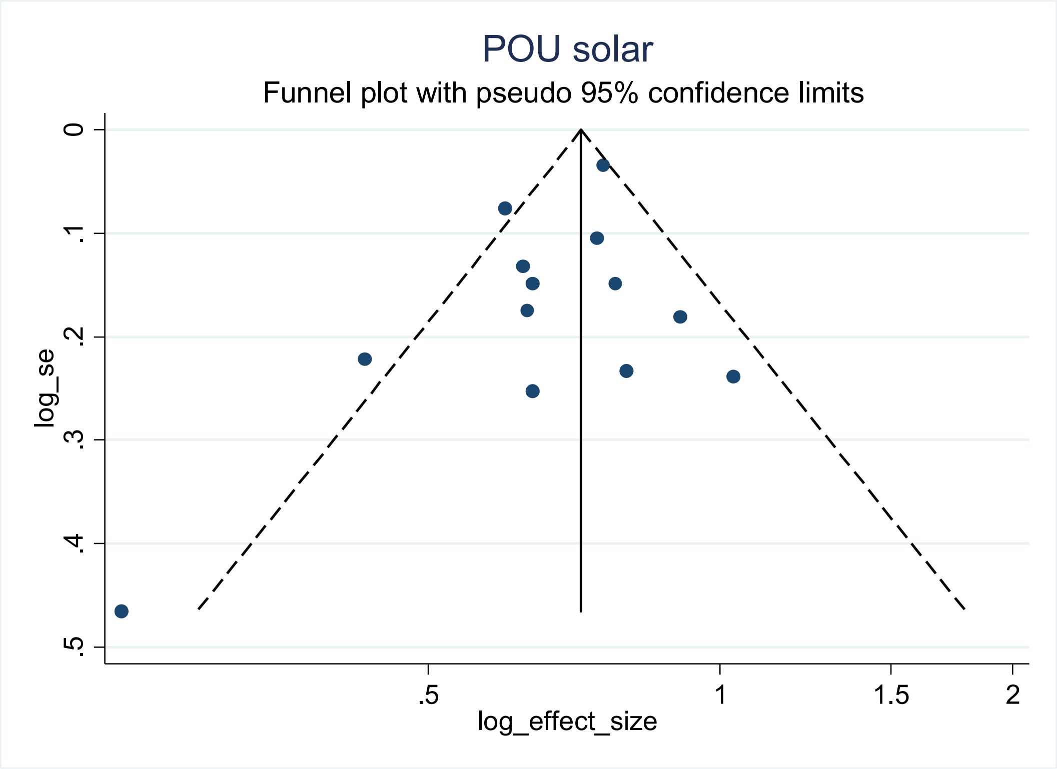

Figure S1·14: Funnel plot of drinking water interventions: point-of-use (POU) solar treatment

Egger test: p = 0·21

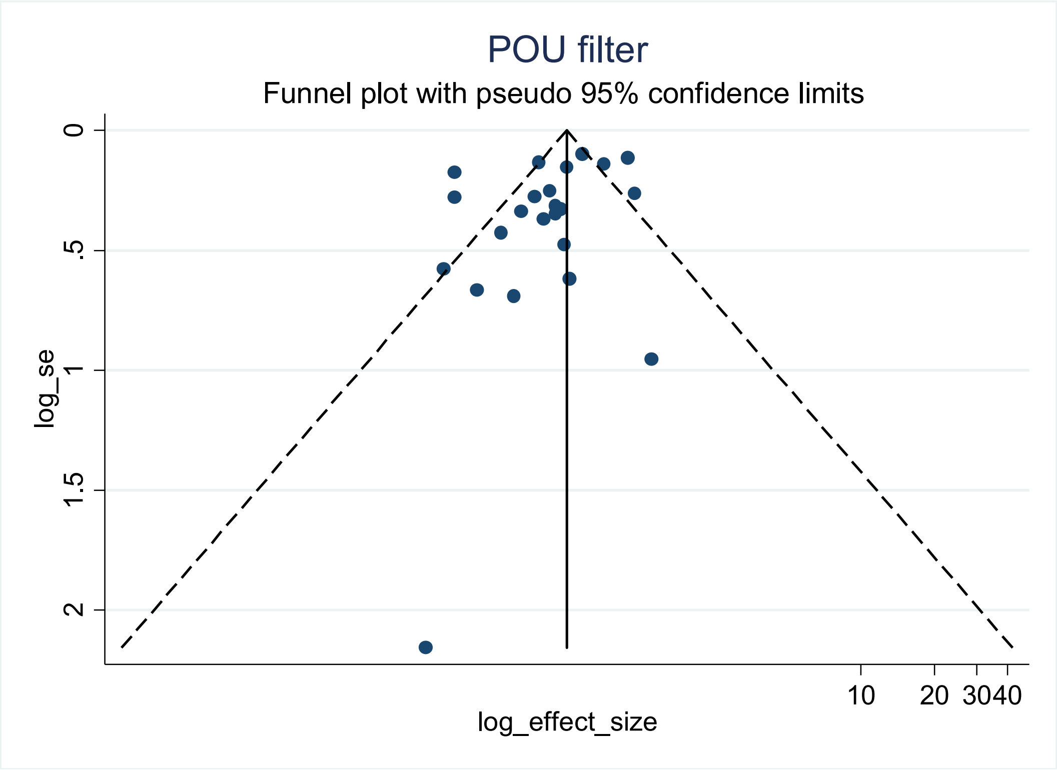

Figure S1·15: Funnel plot of drinking water interventions: point-of-use (POU) filter treatment

Egger test: p = 0·09

Sanitation

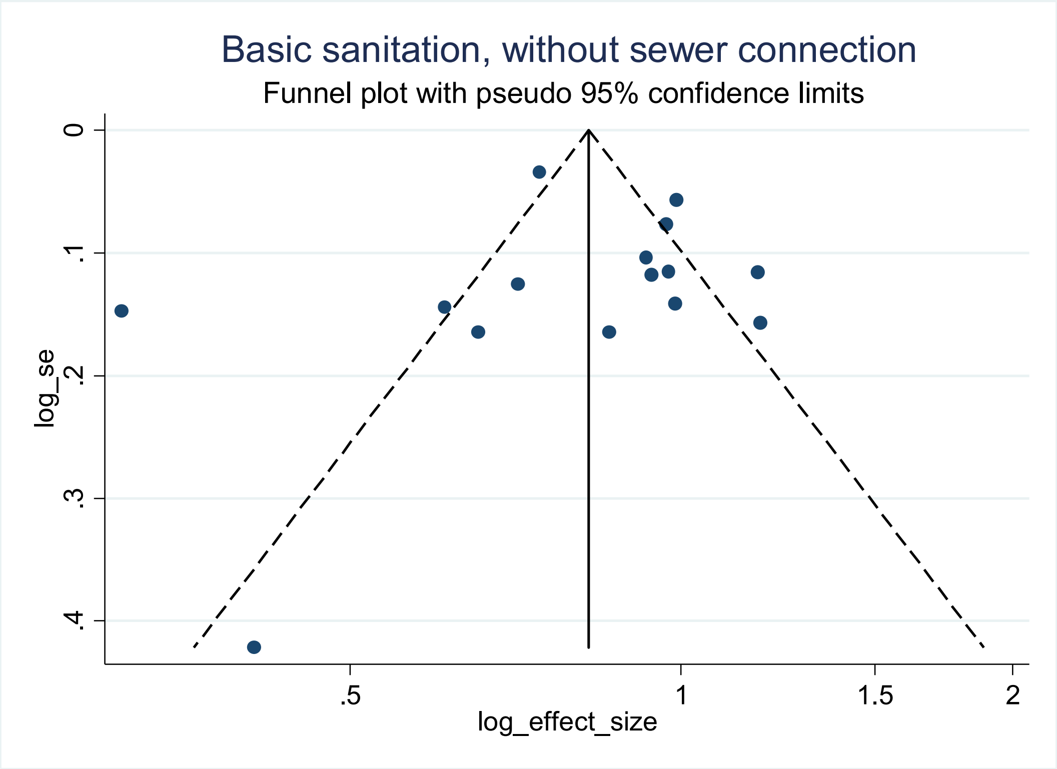

Figure S1·16: Funnel plot of sanitation interventions: basic on-site sanitation, without sewer connection

Egger test: p = 0·99

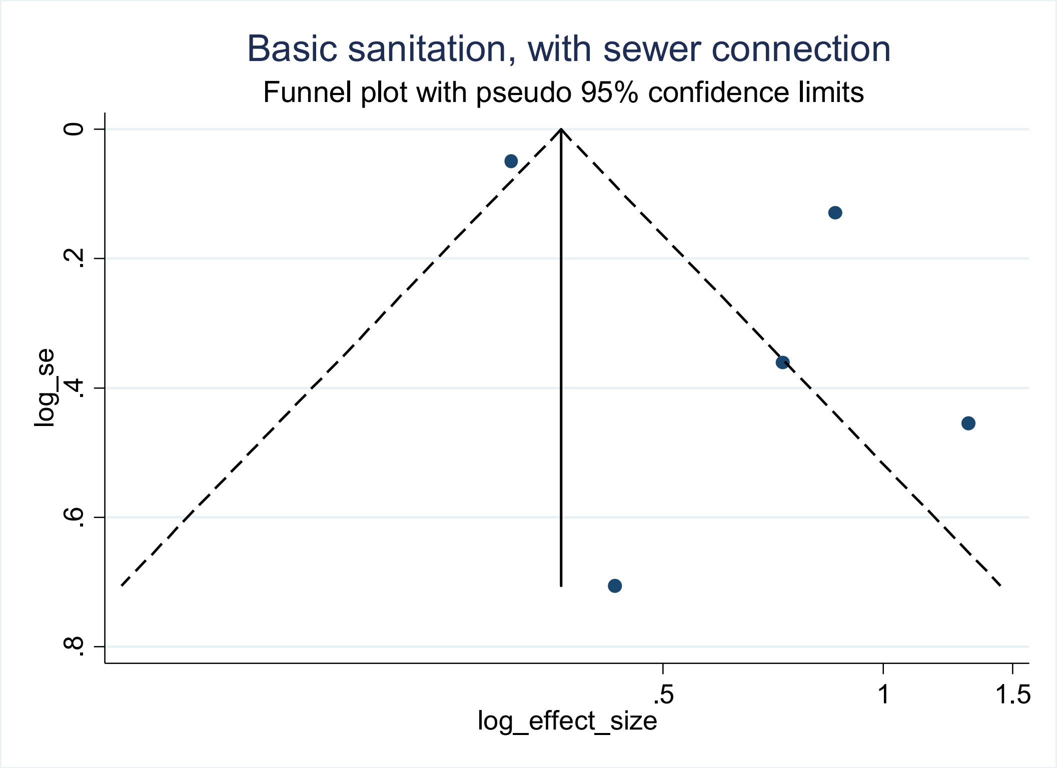

Figure S1·17: Funnel plot of sanitation interventions: basic on-site sanitation, with sewer connection

Hygiene

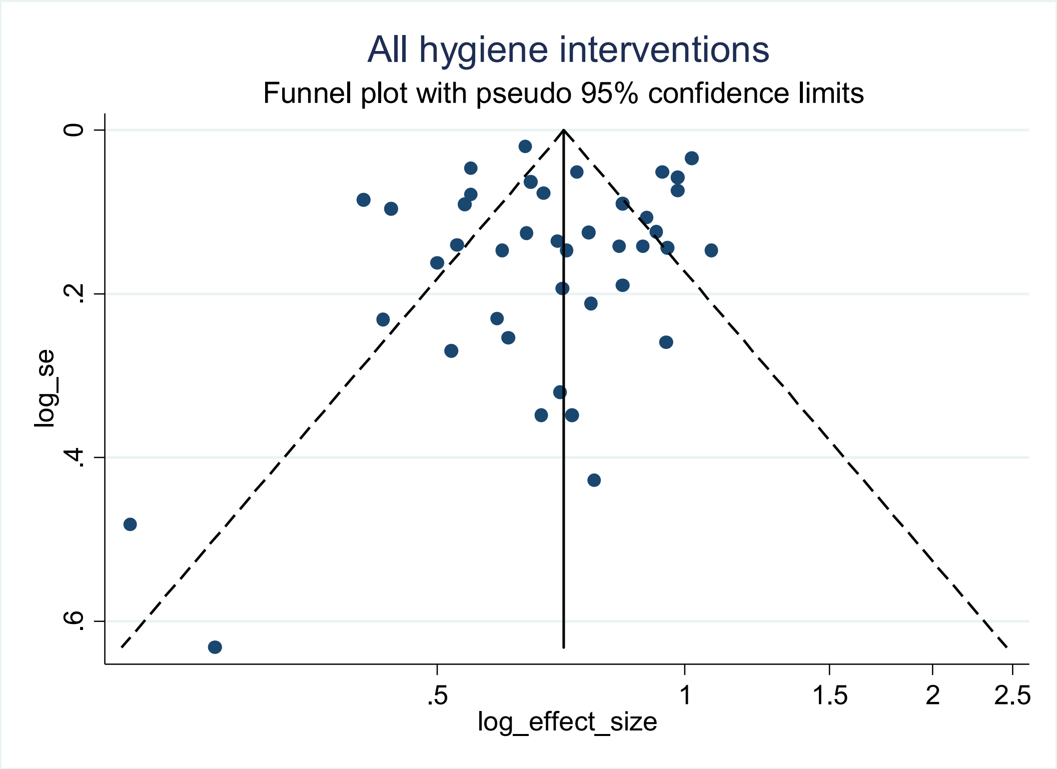

Figure S1·18: Funnel plot of all hygiene interventions

Egger test: p = 0·8

8. Drinking water and sanitation service levels according to the JMP (service ladders)

Definitions are taken ad verbatim from the JMP website[38].

Drinking water

Safely managed drinking water: Drinking water from an improved water source, which is located on premises, available when needed and free from faecal and priority chemical contamination.

Basic drinking water: Drinking water from an improved source, provided collection time is not more than 30 minutes for a roundtrip including queuing.

Limited drinking water: Drinking water from an improved source for which collection time exceeds 30 minutes for a roundtrip including queuing.

Unimproved drinking water: Drinking water from an unprotected dug well or unprotected spring, surface drinking water that is water from a river, dam, lake, pond, stream, canal or irrigation canal.

Improved drinking water sources are those that have the potential to deliver safe water by nature of their design and construction, and include: piped water, boreholes or tubewells, protected dug wells, protected springs, rainwater, and packaged or delivered water. [39]

Sanitation

Safely managed sanitation: Use of improved facilities, which are not shared with other households and where excreta are safely disposed in situ or transported and treated off-site.

Basic sanitation: Use of improved facilities, which are not shared with other households.

Limited sanitation: Use of improved facilities shared between two or more households.

Unimproved sanitation: Use of pit latrines without a slab or platform, hanging latrines or bucket latrines, and open defecation which is the disposal of human faeces in fields, forests, bushes, open bodies of water, beaches and other open spaces or with solid waste.

Improved sanitation facilities are those designed to hygienically separate excreta from human contact, and include: flush/pour flush to piped sewer system, septic tanks or pit latrines; ventilated improved pit latrines, composting toilets or pit latrines with slabs. [40]

Hygiene

Basic handwashing facility: Availability of a handwashing facility on premises with soap and water.

Limited handwashing facility: Availability of a handwashing facility on premises without soap and water.

No facility: No handwashing facility on premises.

Handwashing facilities may be fixed or mobile and include a sink with tap water, buckets with taps, tippy-taps, and jugs or basins designated for handwashing. Soap includes bar soap, liquid soap, powder detergent, and soapy water but does not include ash, soil, sand or other handwashing agents.

9. GRADE assessment

GRADE scores the body of evidence as high, moderate, low or very low certainty evidence and assesses the level of confidence that the estimated effect is close to the true effect [41]. When using GRADE, randomized interventions start as 'high' certainty evidence while observational studies start as 'low' certainty evidence [41]. As we use evidence from both randomized and non-randomized interventions, we decided to start as 'high' certainty evidence when the majority of comparisons for the respective type of WASH intervention were from randomized studies and 'low' otherwise. We then used the five GRADE criteria to potentially downgrade the initial rating: risk of bias in individual studies, inconsistency, indirectness, imprecision and publication bias [41]. Risk of bias was assessed using the average score from the adapted Newcastle-Ottawa scale (Supplementary File 3). An average rounded score of six or higher was considered as no serious risk of bias, three to five as serious risk of bias (downgraded one level) and zero to two very serious risk of bias (downgraded two levels). Inconsistency was assessed through an I^2 measure of heterogeneity in effect sizes of >90% indicating substantial inconsistency (downgraded one level). We chose 90% as the cut-off as complex public health interventions such as WASH interventions usually have greater between-study heterogeneity compared to clinical interventions [42]. Reasons for downgrading one level for indirectness were if the study was not directly able to measure the intervention of interest. In addition, where evidence was available for only one or two settings, the results were considered to have limited generalizability to other settings, and were also downgraded one level for indirectness. We downgraded for imprecision when the pooled RR from meta-analysis included 1 and when the pooled RR was based on few studies and few participants (downgraded one level). For assessing the presence of publication bias we visually inspected funnel plots by intervention type and applied the Egger test for small study effects when there were at least 10 comparisons included in the meta-analysis [43] (Table S1·5, funnel plots in Figures S1·10 to S1·18 with results of Egger test presented below the funnel plots).

Table S1.7: GRADE rating approach by intervention type

	Number of studies	Start-off score[1] (prop. randomized)	Risk of bias (mean NOS)[2]	Indirectness	Inconsistency[3] (I2)	Imprecision[4]	Publication bias	Final GRADE
**Water interventions**								
Improved, not on premises	10	Low (0·2)	-2 (1·6)	0	-1 (98%)	0	0	Very low certainty evidence
Improved, on premises	9	Low (0·11)	-1 (2·9)	0	0 (85%)	-1	0	Very low certainty evidence
Improved, on premises, higher water quality	2	Low (0·5)	-1 (4·5)	-1[6]	0 (76%)	-1	0	Very low certainty evidence
Improved, on premises, continuous supply	1	Low (0)	-1 (3)	- 1[7]	NA – one study[8]	-1	NA – one study[8]	Very low certainty evidence
POU chlorine	25	High (0·84)	-1 (4·1)	0	0 (89%)	0	0	Moderate certainty evidence
POU solar	13	High (0·85)	-1 (3·4)	0	0 (54%)	0	0	Moderate certainty evidence
POU filter	23	High (0·87)	-1 (4·3)	0	0 (80%)	0	0	Moderate certainty evidence
**Sanitation interventions**								
Basic sanitation, without sewer connection	15	High (0·6)	-1 (3·3)	0	0 (86%)	0	0	Moderate certainty evidence
Basic sanitation, with sewer connection	5	Low (0)	-2 (1·6)	0	-1 (94%)	-1	0	Very low certainty evidence

**Hygiene interventions**	41	High (0·8)	-1 (3·1)	0	0 (90%)	0	0	Moderate certainty evidence

[1] When majority of studies (i.e. >50%) is randomized start 'high' otherwise 'low',
[2] downgrade by 1 if mean rounded Newcastle Ottawa Scale (NOS) score between 3-5, downgrade by 2 if mean NOS score between 0-2
[3] Inconsistency: I^2 >90%: -1;
[4] Imprecision: pooled RR include 1 and/or based on few studies/participants: -1;
[6] downgraded by 1 as evidence from two different settings (rural Puerto Rico and urban India). Generalization to other settings severely restricted.
[7] downgraded by 1 as single intervention study in one urban setting in India, not possible to generalize across settings
[8] no implication as already graded "very low quality evidence" and no further downgrading possible

10. Comparison with other reviews

For comparison with the previous WASH systematic review, results presented in Wolf et al. 2018 [1] are directly comparable with findings from the current review.

Table S1.8: Comparison with other reviews

**Other review[a]**	**Intervention type**	**Number of comparisons in other review[a]**	**RR of other review**	**Correspondence intervention type in 2021 review[a]**	**RR of 2021 review[a]**	**Comments**
Clasen et al., 2015	POU filter	18	0·48 (0·38, 0·59)	POU filter	All POU filter studies: 0·51 (0·41, 0·65); POU filter compared to reference of unimproved water source: 0·50 (0·41, 0·60)	Other review last updated in 2014, narrower inclusion criteria, 2021 review includes larger number of comparisons (e.g., 11 vs. 6 for *improved, not on premises* and 8 vs. 0 for *improved on premises*).
	POU solar	4	0·62 (0·42, 0·94)	POU solar	All POU solar: 0·63 (0·50, 0·80); POU solar compared to reference of unimproved water	

					source: 0·69 (0·62, 0·77)	
	POU chlorine	14	0·77 (0·65, 0·91)	POU chlorine	All POU chlorine: 0·69 (0·60, 0·78); POU chlorine compared to reference of unimproved water source: 0·66 (0·56, 0·77)	
	Source-based water improvements	-	Pooled RR not calculated	improved, not on premises	Meta-analysis: 0·76 (0·60, 0·95); meta-regression: 0·81 (0·70, 0·94)	
	Piped water to households	-	No studies identified that met inclusion criteria	improved, on premises	All piped: 0·87 (0·68, 1·12), piped compared to reference of unimproved water source: 0·79 (0·60, 1·03)	
Freeman et al., 2017	All sanitation interventions	16	0·77 (0·66, 0·91)	All sanitation interventions	0·76 (0·61, 0·94)	Pooled odds ratio in Freeman review
Norman et al., 2010	All sewer studies	25	0·70 (0·61, 0·79)	All sewer interventions	0·63 (0·32, 1·26)	Combination of intervention and non-intervention studies in Norman review
	All sewer studies compared to "very poor" sanitation in reference group	7	0·41 (0·27, 0·61)	basic sanitation connected to sewer compared to unimproved/limited sanitation	RR 0·53 (0·30, 0·93)	
Ejemot-Nwadiaro et al., 2021	Handwashing promotion in communities in low- and middle income countries	15	RR 0·71 (0·62, 0·81)	Handwashing promotion in communities in low- and middle income countries and in day-care –facilities and schools in high-income countries	RR 0·70 (0·64, 0·76)	In 2021 review, no evidence for an association of community versus day care or schools and high-income setting versus low- and middle-income setting and risk of diarrhoea

						(consistent with Ejemot review)
	Handwashing promotion in day-care facilities and schools in high-income countries	13	RR 0·70 (0·58, 0·85)		RR 0·70 (0·64, 0·76)	
Global Burden of Diseases, Injuries, and Risk Factors Study (GBD) 2019	Boil/filter water treatment compared to unimproved water source	71 water comparisons in total, subgroup numbers unclear	RR 0·49 (0·42, 0·56)	POU filter	all POU filter studies: 0·51 (0·41, 0·65); POU filter compared to reference of unimproved water source: 0·50 (0·41, 0·60)	review unpublished[c]
	Chlorine/solar water treatment compared to unimproved water source	71 water comparisons in total, subgroup numbers unclear	RR 0·73 (0·67, 0·79)	POU chlorine or POU solar	All POU chlorine: 0·69 (0·60, 0·78); POU chlorine compared to reference of unimproved water source: 0·66 (0·56, 0·77)<br><br>All POU solar: 0·63 (0·50, 0·80); POU solar compared to reference of unimproved water source: 0·69 (0·62, 0·77)	
	Improved water source compared to unimproved water source	71 water comparisons in total, subgroup numbers unclear	RR 0·83 (0·76, 0·91)	Improved, not on premises	Meta-analysis: 0·76 (0·60, 0·95); meta-regression: 0·81 (0·70, 0·94)	
	Piped water source compared to unimproved water source	71 water comparisons in total, subgroup numbers unclear	RR 0·64 (0·56, 0·73)	Improved, on premises (interventions included are only piped to premises)	All piped: 0·87 (0·68, 1·12), piped compared to reference of unimproved water source: 0·79 (0·60, 1·03)	

	high-quality piped water source compared to unimproved water source	71 water comparisons in total, subgroup numbers unclear	RR 0·20 (0·09, 0·49)	Improved, on premises, higher water quality	0·55 (0·24, 1·27)	Meta-analysis of two studies in 2021 review
	high-quality piped water compared to unimproved water source without POU treatment[1]	71 water comparisons in total, subgroup numbers unclear	RR 0·09 (0·04, 0·23)[b]	Improved, on premises, higher water quality compared to reference of unimproved water source	0·48 (0·26, 0·87)	
	improved sanitation compared to unimproved sanitation	16 sanitation comparisons in total, subgroup numbers unclear	RR 0·77 (0·73, 0·81)		All sanitation: 0·76 (0·61, 0·94); basic sanitation without sewer connection compared to limited/unimproved sanitation: 0·79 (0·61, 1·03)	
	Sanitation facilities with sewer connection or septic tank compared to unimproved sanitation	16 sanitation comparisons in total, subgroup numbers unclear	RR 0·31 (0·28, 0·34)	Basic sanitation connected to sewer compared to limited/unimproved sanitation	RR 0·53 (0·30, 0·93)	

[a] "other review" as cited, "2021 review" refers to present review (Wolf et al., 2022)

[b] RR calculated from Table 6 as 1/RR and transposing intervention type and reference level

[c] information from reference: "A meta-analysis by Wolf et al, 2014 [citation] was complemented by a literature review that searched PubMed for related intervention studies post-2014. Search terms used were identical to those provided by Wolf et al, 2014 [..]. Additionally, for GBD 2019, new relative risk evidence was added using an updated version of the 2014 Wolf et al meta-analysis that was published in 2018 [..]."

References

1 Wolf J, Hunter PR, Freeman MC, *et al.* Impact of drinking water, sanitation and hand washing with soap on childhood diarrhoeal disease: updated meta-analysis and meta-regression. *Trop Med Int Health* 2018; **23**: 508–25.

2 Wolf J, Prüss-Ustün A, Cumming O, *et al.* Assessing the impact of drinking-water and sanitation on diarrhoeal disease in low-and middle-income settings: A systematic review and meta-regression. *Trop Med Int Health* 2014; **19**: 928–42.

3 Austin CJ. Investigation of in-house water chlorination and its effectiveness for rural areas of the Gambia. 1993.

4 Boisson S, Stevenson M, Shapiro L, *et al.* Effect of Household-Based Drinking Water Chlorination on Diarrhoea among Children under Five in Orissa, India: A Double-Blind Randomised Placebo-Controlled Trial. *PLoS medicine* 2013; **10**: e1001497.

5 Jain S, Sahanoon OK, Blanton E, *et al.* Sodium Dichloroisocyanurate Tablets for Routine Treatment of Household Drinking Water in Periurban Ghana: A Randomized Controlled Trial. *Am J Trop Med Hyg* 2010; **82**: 16–22.

6 Kirchhoff LV, McClelland KE, Pinho MDC, Araujo JG, De Sousa MA, Guerrant RL. Feasibility and efficacy of in-home water chlorination in rural North-eastern Brazil. *Journal of Hygiene* 1985; **94**: 173–80.

7 Betancourt WQ, Rose JB. Drinking water treatment processes for removal of Cryptosporidium and Giardia. *Veterinary Parasitology* 2004; **126**: 219–34.

8 Hartinger SM, Lanata CF, Hattendorf J, *et al.* Improving household air, drinking water and hygiene in rural Peru: a community-randomized–controlled trial of an integrated environmental home-based intervention package to improve child health. *International journal of epidemiology* 2016; **45**: 2089–99.

9 Reese H, Routray P, Torondel B, *et al.* Assessing longer-term effectiveness of a combined household-level piped water and sanitation intervention on child diarrhoea, acute respiratory infection, soil-transmitted helminth infection and nutritional status: a matched cohort study in rural Odisha, India. *International journal of epidemiology* 2019. DOI:10.1093/ije/dyz157.

10 Lipsitch M, Tchetgen ET, Cohen T. Negative Controls: A Tool for Detecting Confounding and Bias in Observational Studies. *Epidemiology* 2010; **21**: 383–8.

11 Borenstein M, Hedges LV, Higgins JPT, Rothstein HR. Part 5: Complex data structures. In: Introduction to Meta-Analysis, 1st edn. United Kingdom: John Wiley & Sons, Ltd, 2009: 213–45.

12 Luby SP, Agboatwalla M, Painter J, *et al.* Combining drinking water treatment and hand washing for diarrhoea prevention, a cluster randomised controlled trial. *Trop Med Int Health* 2006; **11**: 479–89.

13 Aziz KMA, Hoque BA, Hasan KZ, *et al.* Reduction in diarrhoeal diseases in children in rural Bangladesh by environmental and behavioural modifications. *Transactions of the Royal Society of Tropical Medicine and Hygiene* 1990; **84**: 433–8.

14 Manaseki-Holland S, Manjang B, Hemming K, *et al.* Effects on childhood infections of promoting safe and hygienic complementary-food handling practices through a community-based programme: A cluster randomised controlled trial in a rural area of The Gambia. *PLoS medicine* 2021; **18**: e1003260.

15 Messou E, Sangaré SV, Josseran R, Le CC, Guélain J. Effect of hygiene measures, water sanitation and oral rehydration therapy on diarrhea in children less than five years old in the south of Ivory Coast. *Bulletin de la Societe de pathologie exotique (1990)* 1997; **90**: 44–7.

16 Higgins JPT, Eldridge S, Li T, editors. Chapter 23: Including variants on randomized trials. In: Cochrane Handbook for Systematic Reviews of Interventions. Cochrane, 2021. www.training.cochrane.org/handbook.

17 Schmidt W-P, Arnold BF, Boisson S, *et al.* Epidemiological methods in diarrhoea studies—an update. *Int J Epidemiol* 2011; **40**: 1678–92.

18 Garrett V, Ogutu P, Mabonga P, *et al.* Diarrhoea prevention in a high-risk rural Kenyan population through point-of-use chlorination, safe water storage, sanitation, and rainwater harvesting. *Epidemiology & Infection* 2008; **136**: 1463–71.

19 Kolahi A-A, Rastegarpour A, Sohrabi M-R. The impact of an urban sewerage system on childhood diarrhoea in Tehran, Iran: a concurrent control field trial. *Transactions of the royal society of tropical medicine and hygiene* 2009; **103**: 500–5.

20 Moraes LRS, Cancio JA, Cairncross S, Huttly S. Impact of drainage and sewerage on diarrhoea in poor urban areas in Salvador, Brazil. *Transactions of the Royal Society of Tropical Medicine and Hygiene* 2003; **97**: 153–8.

21 Pradhan M, Rawlings LB. The impact and targeting of social infrastructure investments: Lessons from the Nicaraguan Social Fund. 2002.

22 Walker I, del Cid R, Ordoñez F, Rodríguez F. Ex-post evaluation of the Honduran Social Investment Fund (FHIS 2). *World Bank, Washington, DC* 1999. http://www.esa.hn/pub/FHIS%20Expost%20Evaluation.pdf (accessed March 10, 2014).

23 Arnold BF, Khush RS, Ramaswamy P, *et al.* Causal inference methods to study nonrandomized, preexisting development interventions. *Proceedings of the National Academy of Sciences* 2010; **107**: 22605–10.

24 Briceño B, Coville A, Martinez S. Promoting Handwashing and Sanitation: Evidence from a Large-Scale Randomized Trial in Rural Tanzania. The World Bank, 2015.

25 Clasen T, Boisson S, Routray P, *et al.* Effectiveness of a rural sanitation programme on diarrhoea, soil-transmitted helminth infection, and child malnutrition in Odisha, India: a cluster-randomised trial. *Lancet Glob Health* 2014; **2**: e645-653.

26 Dickinson KL, Patil SR, Pattanayak SK, Poulos C, Yang J-H. Nature's call: impacts of sanitation choices in Orissa, India. *Economic Development and Cultural Change* 2015; **64**: 1–29.

27 Hammer J, Spears D. Village sanitation and child health: effects and external validity in a randomized field experiment in rural India. *Journal of health economics* 2016; **48**: 135–48.

28 Humphrey JH, Mbuya MN, Nitozini R, *et al.* Independent and combined effects of improved water, sanitation, and hygiene, and improved complementary feeding, on child stunting and anaemia in rural Zimbabwe: a cluster-randomised trial. *Lancet Glob Health* 2019; **7**: e132-47.

29 Klasen S, Lechtenfeld T, Meier K, Rieckmann J. Benefits trickling away: The health impact of extending access to piped water and sanitation in urban Yemen. Göttingen: Georg-August-Universität, 2012.

30 Luby SP, Rahman M, Arnold BF, *et al.* Effects of water quality, sanitation, handwashing, and nutritional interventions on diarrhoea and child growth in rural Bangladesh: a cluster randomised controlled trial. *The Lancet Global Health* 2018; **6**: e302–15.

31 Null C, Stewart CP, Pickering AJ, *et al.* Effects of water quality, sanitation, handwashing, and nutritional interventions on diarrhoea and child growth in rural Kenya: a cluster-randomised controlled trial. *The Lancet Global Health* 2018; **6**: e316–29.

32 Patil SR, Arnold BF, Salvatore AL, *et al.* The effect of India's total sanitation campaign on defecation behaviors and child health in rural Madhya Pradesh: a cluster randomized controlled trial. *PLoS Med* 2014; **11**: e1001709.

33 Pickering AJ, Djebbari H, Lopez C, Coulibaly M, Alzua ML. Effect of a community-led sanitation intervention on child diarrhoea and child growth in rural Mali: a cluster-randomised controlled trial. *Lancet Glob Health* 2015; **3**: e701-711.

34 Aiken BA, Stauber CE, Ortiz GM, Sobsey MD. An assessment of continued use and health impact of the concrete biosand filter in Bonao, Dominican Republic. *Am J Trop Med Hyg* 2011; **85**: 309–17.

35 Brown J, Sobsey MD, Loomis D. Local drinking water filters reduce diarrheal disease in Cambodia: a randomized, controlled trial of the ceramic water purifier. *The American journal of tropical medicine and hygiene* 2008; **79**: 394–400.

36 Brown J. Improving Household Drinking Water Quality, Use of Ceramic Water Filters in Cambodia. Water and Sanitation Program, UNICEF, 2007 https://www.wsp.org/sites/wsp/files/publications/926200724252_eap_cambodia_filter.pdf (accessed June 21, 2021).

37 Harshfield E, Lantagne D, Turbes A, Null C. Evaluating the sustained health impact of household chlorination of drinking water in rural Haiti. *Am J Trop Med Hyg* 2012; **87**: 786–95.

38 WHO, UNICEF. Home | JMP. undated. https://washdata.org/ (accessed Sept 9, 2019).

39 WHO, UNICEF. Monitoring: Drinking water. JMP. https://washdata.org/monitoring/drinking-water (accessed Sept 9, 2019).

40 WHO, UNICEF. Monitoring: Sanitation. JMP. https://washdata.org/monitoring/sanitation (accessed Sept 9, 2019).

41 Guyatt G, Oxman AD, Akl EA, *et al.* GRADE guidelines: 1. Introduction—GRADE evidence profiles and summary of findings tables. *Journal of Clinical Epidemiology* 2011; **64**: 383–94.

42 Murad MH, Almasri J, Alsawas M, Farah W. Grading the quality of evidence in complex interventions: a guide for evidence-based practitioners. *BMJ Evidence-Based Medicine* 2017; **22**: 20–2.

43 Page MJ, Higgins JPT, Sterne JAC. Chapter 13: Assessing risk of bias due to missing results in a synthesis. In: Cochrane Handbook for Systematic Reviews of Interventions, 6.2. Cochrane, 2021. https://training.cochrane.org/handbook/current/chapter-13 (accessed June 22, 2021).
